# Multi-Omics Comparison of the Spontaneous Diabetes Mellitus and Diet-Induced Prediabetic Macaque Models

**DOI:** 10.3389/fphar.2021.784231

**Published:** 2021-11-22

**Authors:** Zhu Yang, Dianqiang Yang, Fancheng Tan, Chi Wai Wong, James Y. Yang, Da Zhou, Zongwei Cai, Shu-Hai Lin

**Affiliations:** ^1^ State Key Laboratory of Cellular Stress Biology, School of Life Sciences, Xiamen University, Xiamen, China; ^2^ State Key Laboratory of Environmental and Biological Analysis, Department of Chemistry, Hong Kong Baptist University, Hong Kong, China; ^3^ Guangzhou Huazhen Biosciences Co., Ltd., Guangzhou, China; ^4^ School of Mathematical Sciences, Xiamen University, Xiamen, China

**Keywords:** spontaneous diabetes mellitus, non-human primates, cynomolgus monkey (*Macaca fascicularis*), metabolomics, transcriptomics, proteomics

## Abstract

The prevalence of diabetes mellitus has been increasing for decades worldwide. To develop safe and potent therapeutics, animal models contribute a lot to the studies of the mechanisms underlying its pathogenesis. Dietary induction using is a well-accepted protocol in generating insulin resistance and diabetes models. In the present study, we reported the multi-omics profiling of the liver and sera from both peripheral blood and hepatic portal vein blood from *Macaca fascicularis* that spontaneously developed Type-2 diabetes mellitus with a chow diet (sDM). The other two groups of the monkeys fed with chow diet and high-fat high-sugar (HFHS) diet, respectively, were included for comparison. Analyses of various omics datasets revealed the alterations of high consistency. Between the sDM and HFHS monkeys, both the similar and unique alterations in the lipid metabolism have been demonstrated from metabolomic, transcriptomic, and proteomic data repeatedly. The comparison of the proteome and transcriptome confirmed the involvement of fatty acid binding protein 4 (FABP4) in the diet-induced pathogenesis of diabetes in macaques. Furthermore, the commonly changed genes between spontaneous diabetes and HFHS diet-induced prediabetes suggested that the alterations in the intra- and extracellular structural proteins and cell migration in the liver might mediate the HFHS diet induction of diabetes mellitus.

## Introduction

Diabetes mellitus (DM) is a metabolic malfunction, characterized by a prolonged high blood glucose level, the prevalence of which has been steadily elevating in the past decades (Martins, 2014; Zhang et al., 2020). DM occurs when the pancreas produces insufficient insulin, and/or the body tissues, such as the liver, resist the actions of insulin (Martins, 2018a). Animal models, as in the studies on many other human diseases, have been wildly applied to the investigations of this metabolic disorder affecting multiple organ systems (Martins, 2017; Kleinert et al., 2018; Backman et al., 2019). The animals that developed diabetes mellitus spontaneously provided important insights into the molecular and cellular pathology of DM (Yasuda et al., 1988; Bauer et al., 2011; Harwood et al., 2012; Wang et al., 2013). Because animals of other orders do not fully recapitulate metabolic changes of primates, non-human primates (NHPs) are of value in DM study with genetic and physiological similarity to humans (Yasuda et al., 1988; Hansen, 2010; Bauer et al., 2011; Harwood et al., 2012; Pound et al., 2014; Havel et al., 2017; Lei et al., 2020; Cox et al., 2021). With the datasets representing the cellular and molecular alterations at various levels in NHPs of spontaneous DM (sDM), researchers can project the data from clinical and experimental studies to the picture of the correlated metabolic and gene expression profiles.

Increased demands in comprehensive and quantitative profiling at the molecular levels, along with the improvements in analytical biotechnology lead to the rapid development of the “omics” research (Karczewski and Snyder, 2018; Colli et al., 2020). All branches of omics, such as metabolomics, transcriptomics, and proteomics, identify and quantify hundreds to tens of thousands of targets in a high-throughput manner, providing thorough snapshots of cellular molecules (Kulkarni et al., 2019; Mistry et al., 2019; Damiani et al., 2020; Noor et al., 2021). The metabolome represents the molecular fingerprint left behind cellular processes, generating a link between cellular regulation and phenotypes (Pearson, 2007; Holmes et al., 2008). Because of its high tolerance to volatility and wider molecule coverage, liquid chromatography-tandem mass spectrometry (LC-MS/MS) is the technique of choice in metabolomics studies (Ren et al., 2018). It has been demonstrated that the biochemical process of DM results in significant signatures in the metabolome (Xu et al., 2013; Monnerie et al., 2020; Sun et al., 2020). The compounds correlated with the risk of DM, either positively or negatively, have been documented from both clinical and experimental investigations (Mora-Ortiz et al., 2019; Fikri et al., 2020; Sun et al., 2020). Transcriptomics, which analyses gene expression profiling, is a commonly used tool for investigating the regulation in response to internal and external cues. It has been well accepted that genetics and environments are both risk factors of DM (Murea et al., 2012; Tremblay and Hamet, 2019). The RNA sequencing (RNA-Seq) utilizing the next-generation sequencing technology allows the quantification of virtually the whole transcriptome in a sample (Stark et al., 2019). Previous work has shown that transcriptomics provides the direct links between genotype and phenotype in DM (Jenkinson et al., 2016; Christodoulou et al., 2019). Moreover, proteomics profiles the end products of gene regulation, and a combined transcriptomic and proteomic datasets would enable the portraits of the regulatory changes in gene expression (Liu et al., 2016; Kühl et al., 2017; Dai et al., 2018). Shotgun proteomics using the label-free quantification (LFQ) strategy, which is in principle the most easily and widely applicable, offers reliable quantification results with the development of experimental approaches and data-procession tools.

The liver plays a major role in the metabolism of carbohydrates and has been well considered as an essential organ in DM (Meshkani and Adeli, 2009; Macdonald, 2016; Tilg et al., 2017; De Silva et al., 2019). Insulin produced from the beta cells first reaches the liver *via* the hepatic portal vein, regulating the storage and release of glucose. The liver, as the reservoir of sugar, is one of the primary tissues developing insulin resistance in DM patients. A mount of studies has revealed the abnormal gene expression and metabolic alterations in the liver caused by DM (Tilg et al., 2017; Li et al., 2019). It has long been accepted that insulin resistance attributes, at least partially, to the higher intake of dietary fat and sugar. Animal models of diet-induced insulin resistance are of validity in the DM study, which has been widely applied to investigate the pathogenesis of insulin resistance as well as DM (Kleinert et al., 2018). Furthermore, having spatial information, which is measured from the various types of tissues and organs, is generally more valuable to explain the changes involved in the processes of disease development. Peripheral blood (PB) is one of the most accessible samples clinically. DM, as a disorder of multiple organ systems, significantly changes the molecules carried in PB, which has been discovered from many studies (O'Kell et al., 2017; Zheng et al., 2021a). The hepatic portal vein blood (HPVB) carries not only metabolites from the abdominal organs but also the important proteins/peptides like insulin from the pancreas to the liver (Edgerton et al., 2019). The available data, albeit limited, illustrate that it variations between the DM cases and the healthy controls.

**FIGURE 1:**
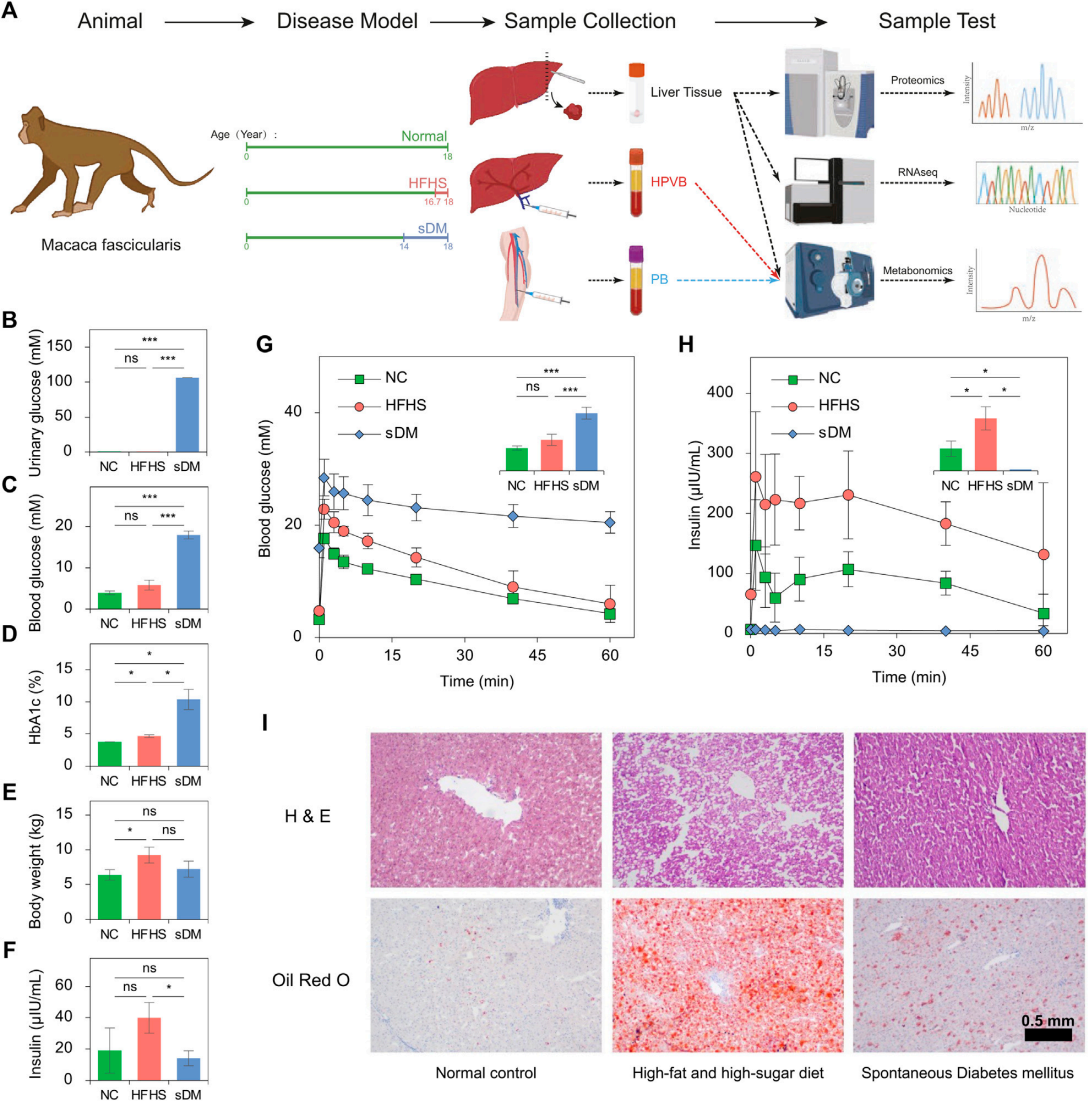
The design of the experiments. **(A)** Experimental workflow. Three groups of *Macaca fascicularis* were sacrificed and their liver, PB, and HPVB were collected for metabolomic, transcriptomic, and proteomic profiling. **(B–F)** The comparison of urinary glucose **(B)**, blood glucose **(C)**, HbA1c **(D)**, bodyweight **(E)**, and insulin **(F)** between the groups. **(G)** and **(H)** The changes in blood glucose **(G)** and insulin **(H)** during IVGTT. **(I)** The representatives of the histochemical staining results of the macaque livers from the three groups.

In the present study, we diagnosed spontaneous type 2 DM (sDM) on three cynomolgus monkeys (*Macaca fascicularis*) of about 15 years old (Figure 1A). To investigate the hepatic changes caused by extra calories related to the development of insulin resistance and diabetes, we also enlisted another three cynomolgus macaques who developed prediabetic symptoms after 15-months feeding of high-fat and high-sugar (HFHS) diet. About 3 years after the diagnosis, the liver tissue, PB, and HPVB samples were collected from both groups of macaques and chow diet-fed normal controls (NC) of a similar age (Figure 1A). We sampled the metabolome, transcriptome, and proteome from the liver of each individual. Highly consistent results were found from multi-omics data. Analysis of metabolomic, transcriptomic, and proteomic alone and joint revealed that both the diabetes conditions and the HFHS diet caused the alterations in lipid metabolism. Also, we found that the commonly changed genes of these two groups of macaques suggested that the hepatic alterations in the extracellular matrix and cell migration might function importantly at the early stage of the HFHS diet-induced diabetes mellitus. These multi-omics datasets from the liver and blood are also a valuable resource for comparing results with other experimental or clinical studies.

## Materials and Methods

### Macaques and Growth Conditions

Male *Macaca fascicularis* of about 18 years old were bought from Huazhen Biosciences Co., Ltd. (Guangzhou, China). All macaques were born from 16^th^ August 2001 to 11^th^ February 2004 (Supplementary Table S1) and grown in the animal rooms maintained at 16 ∼ 26°C and 40–70% room humidity on a 12-h/12-h light-dark cycle. All monkeys were housed individually in standard stainless steel cages (80 cm × 80 cm × 85 cm) and the assigned diets were supplemented twice a day at 8:00 (100 g) and 16:00 (150 g), plus 100 g of apple at 12:00. The animals were allowed to access water freely. The macaques of both NC and sDM groups were fed with the normal food (19.26% protein, 5% fat, no sugar, and no cholesterol), whereas the HFHS monkeys were grown up on the same diet until switching to the high-fat and high-sugar (HFHS) food (≧ 30% sugar, ≧ 15% fat, ≧ 10.5% protein, and ≧ 0.5% cholesterol) on 1^st^ March 2019 (Figure 1A). After 15 months of food change, blood and urine samples were collected from all macaques after 12–14 h of fasting for examination and the intravenous glucose tolerance test (IVGTT) was conducted. The routine hematological examination was performed on the Hematology Analyzer pocH-100iV (Sysmex, Kobe, Japan). Insulin was quantified using the Cobas E411 Analyzer (Roche, Basel, Switzerland), and other blood biochemical analyses, including the measurement of blood sugar, were done by the Cobas C311 Analyzer (Roche, Basel, Switzerland). Urinary analysis was done in the local hospital Guangzhou Conghua District Hospital of Traditional Chinese Medicine. IVGTT was done in the morning after 12–14 h of fasting. In IVGTT, 50% (w/v) glucose solution was injected into the limb vein of the anesthetized monkeys (1 ml/kg body weight) immediately after blood collection (0 min) from another limb. Then the blood samples were collected after 1, 3, 5, 10, 20, 40, and 60 min for both sugar and insulin analysis.

All macaques were then sacrificed *via* overdose anesthesia at about 18 years old, or 1.25 years after the switch of foods (Figure 1A; Supplementary Table S1). In brief, the animals were fasted for more than 12 h before euthanasia and transferred to the operating room with no other experimental animals present. Ketamine hydrochloride was injected intramuscularly at a dose of 10–25 mg/kg, followed by the intravenous injection of 4% sodium pentobarbital solution (100 mg/kg) after animal stabilization. Two veterinarians checked and confirmed the death of the monkey independently before the dissection. The liver, PB, and HPVB samples were collected by veterinarians. Both blood samples were centrifuged at 3,000 rpm for 10 min at 4°C immediately to separate sera. All samples were frozen in liquid nitrogen and stored at −80°C until use. The study protocol received prior approval (license number 2020025) from the Institutional Animal Care and Use Committee of Guangzhou Huazhen Biosciences.

### Metabolite Extraction

The hydrophilic and hydrophobic compounds were extracted using methanol/water and MTBE/methanol/water solvent systems, respectively. Samples were first thawed on ice. To extract hydrophilic metabolites from the tissue samples, 1 ml of methanol/water (7:3, v/v) was added to 50 mg of the liver, and homogenized with steel balls for 3 min at 30 Hz, followed by 1 min of a vortex. The homogenate was then centrifuged at 12,000 rpm for 10 min at 4°C to collect the supernatant. Hydrophobic compounds were extracted from another 50 mg using a slightly modified protocol. Briefly, homogenization was done with 1 ml of MTBE/methanol (10:3, v/v) and 100 µL of water was mixed with the homogenate to extract before centrifugation. For the sera of PB and HPVB, 3 volumes (v/v) of methanol and a mixture of MTBE and methanol (10:3, v/v) were whirled with the serum samples for 3 min, followed by centrifugation at 12,000 rpm for 10 min at 4°C. All collected supernatants were dried and store at −80°C until LC-MS/MS analysis. Internal standards were dissolved in the solvents before extraction.

### Protein Extraction and Trypsin Digestion

The tissue sample was ground into a powder in liquid nitrogen and mixed with 4 volumes (v/w) of lysis buffer (8 M urea and 1% Protease Inhibitor Cocktail). The mixture was sonicated three times (30 s each time, 2 s gap) on ice using a high-intensity ultrasonic processor (Scientz, Ningbo, China) and centrifuged at 12,000 ×g for 10 min at 4°C. The supernatant was collected and the protein concentration was determined with a BCA kit following the manufacturer’s protocol.

After that, trichloroacetic acid (TCA) was added to the same volume of each sample to a final concentration of 20%. After 1 min vortex, the samples were placed still at 4°C for 2 h, followed by centrifugation at 4,500 ×g for 5 min. The protein pellets were washed with pre-cooled acetone three times and completed dried with a nitrogen stream. The dried pellets were subsequently resuspended in 200 ml of TEAB with ultrasonication. Trypsin was then added at a 1:50 (trypsin:protein, w/w) ratio for digestion overnight. The tryptic peptide solution was reduced with 5 mM dithiothreitol (DTT) for 30 min at 56°C, followed by 15 min of alkylation with 11 mM iodoacetamide (IAA) at room temperature in darkness. The peptides were then separated with C18 cartridges (Waters, Milford, MA, United States) and subjected to LC-MS/MS analysis.

### Total RNA Extraction

Total RNA was extracted using Trizol (Invitrogen, Carlsbad, CA, United States) according to the manufacturer’s instructions. In Brief, about 60 mg of tissues were ground into a powder in liquid nitrogen, and homogenized in the RNA extraction buffer for 2 min, followed by resting horizontally for 5 min. The mix was then centrifuged for 5 min with 12,000 ×g at 4°C, and the supernatant was transferred into a new tube with 0.3 ml chloroform/isoamyl alcohol (24:1). The mix was shacked vigorously for 15 s and centrifuged at 12,000 ×g for 10 min at 4°C. The upper aqueous phase was transferred into a new tube and mixed with an equal volume of isopropyl alcohol, followed by centrifugation at 13,600 rpm for 20 min at 4°C. Then the RNA pellet was washed twice with 1 ml 75% ethanol and centrifuged at 13,600 rpm for 3 min at 4°C to desert the residual ethanol. After 5–10 min of air dry in a biosafety cabinet, the RNA pellet was dissolved in 25–100 µL of DEPC-treated water, followed by qualification and quantification using a NanoDrop and Agilent 2100 bioanalyzer (Thermo Fisher Scientific, MA, United States), respectively.

### Targeted Metabolomics and Lipidomics by Liquid Chromatography-Tandem Mass Spectrometry

Both hydrophilic and hydrophobic extracts were analyzed using a UPLC-ESI-MS/MS system (UPLC, Shim-pack UFLC SHIMADZU CBM A system, SHIMADZU, Japan; MS, QTRAP® System, Sciex, Washington, United States). ACQUITY UPLC HSS T3 C18 column (1.8 µm, 2.1 mm*100 mm, Waters, Milford, MA, United States) was used for UPLC, working with the following parameters: the Column temperature of 40°C, a flow rate of 0.4 ml/min, and injection volume of 2 μL. The analysis of hydrophilic metabolites used water containing 0.1% formic acid (A) and acetonitrile containing 0.1% formic acid (B) as mobile phases, with a gradient program (V/V) as follows: 95:5 at 0 min, 10:90 at 11.0 min, 10:90 at 12.0 min, 95:5 at 12.1 min, and 95:5 at 14.0 min. For lipidomic analysis, the samples were injected onto a Thermo C30 column (2.6 μm, 2.1 mm × 100 mm). Mobile phase was composed of acetonitrile/water (60/40, v/v) containing 0.04% acetic acid and 5 mmol/L ammonium formate (A) and acetonitrile/isopropanol (10/90, v/v) containing 0.04% acetic acid and 5 mmol/L ammonium formate (B). The gradient program (V/V) was as follows: 80:20 at 0 min, 50:50 at 3.0 min, 35:65 at 5.0 min, 25:75 at 9.0 min, 10:90 at 15.5 min, and 80:20 at 16.0 min. The metabolome and lipidome were measured on a mass spectrometric system with triple quadrupole (QqQ) scans combined with LIT scans in both positive and negative ion modes under the control of Analyst 1.6.3 software (Sciex, Washington, United States). The ion spray voltage (IS) of the ESI source was 5500 and −4500 V for positive and negative modes, respectively. Source temperature was set at 500°C, ion source gas I (GSI), gas II (GSII), curtain gas (CUR) at 55, 60, and 25.0 psi, respectively, and collision gas (CAD) high. Instrument tuning and mass calibration in QqQ and LIT modes were performed with 10 and 100 μmol/L polypropylene glycol solutions, respectively. Specific sets of multiple reaction monitoring (MRM) transitions of various periods of retention time were monitored according to an in-house library of metabolites.

### Nano-Liquid Chromatography-Tandem Mass Spectrometry Analysis of Tryptic Peptides

NanoLC-MS/MS was performed by coupling a NanoElute and timsTOF Pro (Bruker, Billerica, MA, United States). The tryptic peptide sample was dissolved in 0.1% formic acid and 2% acetonitrile (solvent A) and directly loaded onto the NanoLC-MS/MS platform. A constant flow rate of 450 nL/min was used for the LC system and the solvent B of mobile phase was 0.1% formic acid in acetonitrile, with a gradient from 4 to 22% in 0–70 min, 22–30% in 70–84 min, 30–80% in 84–87 min, and holding at 80% for the last 3 min. The peptides were subjected to a glass capillary ion source with an electrospray voltage of 2.0 kV for tandem mass spectrometry analysis. The data were collected in parallel accumulation–serial fragmentation (PASEF) mode in which one MS full scan followed by 10 MS/MS scans with 30 s dynamic exclusion was used. Both the precursor ions and fragment ions were analyzed with high-resolution TOF. The peptide precursors of changes from +1 to +5 were detected in a scan range from *m/z* 100 to 1700.

### Construction of mRNA Library and RNA-Seq

Oligo (dT)-attached magnetic beads were applied to purify mRNA from the total RNA. Purified mRNA was fragmented into small pieces with fragment buffer. Then the first-strand cDNA was generated using random hexamer-primed reverse transcription, and the second-strand cDNA was synthesized. A-Tailing Mix and RNA Index Adapters were then added by incubation. The cDNA fragments were amplified by PCR, followed by purification by Ampure XP Beads, and then dissolved in EB solution. The product was validated on an Agilent Technologies 2100 bioanalyzer for quality control. The double-stranded PCR products were heated to denature and circularized by the splint oligo sequence to construct the final library. The final library was amplified with phi29 to make a DNA nanoball (DNB) which had more than 300 copies of one molecular. After that, DNBs were loaded into the patterned nanoarray and single end 50 base reads were generated on the BGIseq500 platform (BGI, Shenzhen, China).

### Raw Data Processing

Integration and correction of the peak areas corresponding to the targeted metabolites from the LC-MRM-MS/MS data were done with MulitQuant (version 3.0, Sciex, Washington, United States), followed by normalization against the total peak areas measured from each sample. The Automatic method of MultiQuent was used with the parameters specified as following: Gaussian smooth width: 0 points; RT half window: 30 s; min peak width: 2 points; min peak height: 800; noise percentage: 70.0%; baseline sub window: 2 min; peak splitting: 2 points; RT tolerance: 0.2 min.

The LC-MS/MS data for proteomics were identified and quantified using the MaxQuant search engine (Ver 1.6.6.0). MS2 were searched against the *Macaca fascicularis* (UP000233100_9541, 46,259 sequences) from the Uniprot database (https://www.uniprot.org/) concatenating with the contaminants database. Target-decay search strategy was applied with decoy databases of reversed sequences. The search parameters were specified as following: cleavage enzyme, Trypsin/P; missing cleavages, up to 2; the mass tolerances for precursor ions, 20 ppm in both the first search and the main search; the mass tolerance for fragment ions, 20 ppm; fixed modification, Carbamidomethyl on cysteine; variable modifications, acetylation at protein N-terminus, oxidation on methionine, and deamidation on both N-terminus and glutamine. False discovery rate, or say FDR, for the peptide-spectrum match (PSM) and protein identification were both 1%. The quantification method was set as LFQ.

After filtering the low-quality reads with SOAPnuke (Ver 1.5.2), sequencing alignment and mapping to the *Macaca fascicularis* genome (GCF_000364345.1) from NCBI (https://www.ncbi.nlm.nih.gov) were done by Bowtie (Ver 2.3.4.3) using the --sensitive preset and HISAT2 (Ver 2.1.0) with the default setting, respectively. The program RSEM (Ver 1.3.1) was then used to quantify the reads and the gene expression level was calculated by the FPKM method. The differentially expressed genes were given by DESeq2 according to *q*-value = 0.05.

### Data Analysis

Student’s t-test and calculation of Pearson correlation coefficients were done in R using the STATS package (v3.6.2). Orthogonal partial least squares discriminant analysis (OPLS-DA) was implemented using the R package ROPLS (v1.20.0), and the variables of log2 ratio outside ±0.6 and variables importance in projection (VIP) > 1 were considered as significantly changed variables. Principal component analysis (PCA) was performed by MetaboAnalyst5.0 (https://www.metaboanalyst.ca) without filtering. The data were first normalized by sum across each sample, then z-scored for each metabolite (i.e., centralized by mean and divided by the standard deviation) before PCA. Gene Ontology (GO) and pathway enrichment were analyzed through the GO Consortium (http://geneontology.org) and KEGG websites (https://www.genome.jp/kegg/), respectively.

## Results

### Experimental Design

Type 2 DM symptom was found in three macaques when they were about 15 years old. We conducted IVGTT on the monkeys and found their ability to secret insulin in response to glucose was significantly reduced compared to age-matched normal monkeys. In addition to the sDM group consisting of these three, other six carb-eating macaques of a similar age and housed at the same farm were recruited as the control in the study. A random half (three ones) of these control macaques were kept growing under the unchanged conditions, termed as normal control (NC). The other three have been fed with the HFHS diet for 1.25 years before sacrifice (Figure 1A), which was named the HFHS group. To profile the health conditions of each individual in detail, we have comprehensively performed clinical tests on each crab-eating macaque (*Macaca fascicularis*) **(** Supplementary Table S1)**.** The diabetes of the sDM monkey was characterized by the highest blood sugar, urine sugar, and glycated hemoglobin (HbA1c) levels (Table 1). As compared with the NC group, the sDM monkeys had dramatically increased urine sugar (677 fold, Figure 1B), and higher levels of both fasting blood sugar (4.6 fold, Figure 1C) and HbA1c (2.7 fold, Figure 1D). The average values of these three indicators of the HFHS ones were also higher than that of the NC group (2.8, 1.5, and 1.2 fold rise in urine sugar, blood sugar, and HbA1c, respectively) although only the increase of HbA1c in the HFHS group showed significantly (Figure 1D). The HFHS-caused alterations in the blood (*p* = 0.06) and urine (*p* = 0.1) sugars had larger *p*-values (Table 1), partially due to the limited number of subjects in each group. According to the definition from the American Diabetes Association, the criteria for diabetic humans are fasting blood glucose ≥7 mM and HbA1c ≥ 6.5% (American Diabetes Association, 2018). The two values of the sDM macaques (17.9 ± 1.0 mM fasting blood glucose and 10.4 ± 1.6% HbA1c) were much higher than these standards although both levels of NHPs are typically lower than humans (Lei et al., 2020). The HFHS group, on the other hand, had a fasting blood glucose of 5.8 ± 1.2 mM and an HbA1c level of 4.7 ± 0.2%, lower than but very close to the boundaries. Furthermore, the HFHS group showed a significant increase in body weight but that of sDM macaques were similar to the normal ones (Figure 1E). Over 1 year of the HFHS feeding seemed to result in a level of insulin significantly higher than that observed in the sDM monkeys (Figure 1F). IVGTT was applied before sacrifice to measure the response of the monkeys to blood glucose. As a result, escalated circulating glucose failed to induce the production of insulin in all sDM macaques, resulting in a prolonged last of high blood glucose (Figures 1G,H). These results indicated that the sDM individuals were at the later stages of type 2 DM with significantly reduced insulin secretion. The HFHS macaques showed enhanced secretion of insulin, but glucose metabolism was still slower than that of the normal ones, suggesting insulin resistance in these monkeys. The Oil Red O Staining of the liver tissues demonstrated the accumulation of lipids in the liver of the HFHS-fed monkeys. Although much less than that of the HFHS group, more lipid droplets were formatted in the liver of sDM macaques, as compared with that of the NC group (Figure 1I). These test results indicated that the three monkeys in the sDM group were suffering from diabetes mellitus, whereas in the HFHS group, the subjects who had developed some lesions similar to that of DM symptoms were prediabetes. We, therefore, profiled both hydrophilic and hydrophobic metabolites in the liver, PB, and HPVB of all nine individuals (Figure 1A). In parallel, we also extracted RNA and protein from all liver samples and measured transcriptomic and proteomic abundance profiles using RNA-seq and shotgun proteomics approaches, respectively (Figure 1A).

**TABLE 1:** The clinical tests of all three groups of macaque.

	Mean (± SD)^a^			Ratio (*p*-value)^b^		
	NC	HFHS	sDM	HFHS/NC	sDM/NC	sDM/HFHS
Age (year)	17.7 (±1.2)	18.2 (±0.7)	18.2 (±1)	1.03 (0.5	1.03 (0.6)	1 (1)
Bodyweight (kg)	6.4 (±0.8)	9.3 (±1.1)	7.2 (±1.2)	1.44 (***0.02***)	1.13 (0.4)	0.78 (0.1)
Blood						
Blood glucose (mM)	3.9 (±0.4)	5.8 (±1.2)	17.9 (±1)	1.48 (0.06)	4.56 (***2×10*** ^***−5***^)	3.09 (***2×10*** ^***−4***^)
Insulin (μIU/ml)	19.1 (±14.4)	39.9 (±9.8)	14.2 (±4.8)	2.09 (0.1)	0.74 (0.6)	0.35 (***0.01***)
HbA1c (%)	3.8 (±0)	4.7 (±0.2)	10.4 (±1.6)	1.23 (***0.02***)	2.73 (***0.02***)	2.22 (***0.02***)
LDL (mM) ^c^	0.8 (±0.2)	9.3 (±3.6)	2.6 (±1.9)	11.12 (0.05)	3.07 (0.24)	0.28 (***0.04***)
CREA (μM) ^c^	72.7 (±9.7)	88.8 (±10.4)	59.6 (±5.1)	1.22 (0.1)	0.82 (0.1)	0.67 (***0.01***)
MCH (10^-1^pg) ^c^	23.9 (±0.3)	22.8 (±0.5)	23.3 (±1.6)	0.95 (***0.04***)	0.98 (0.6)	1.02 (0.6)
Na^+^ (mM)	151.5 (±2)	150.1 (±8.6)	147.5 (±1.1)	0.99 (0.8)	0.97 (***0.04***)	0.98 (0.6)
Cl^−^ (mM)	109.5 (±1.7)	113.1 (±3.7)	106.5 (±1.9)	1.03 (0.2)	0.97 (0.1)	0.94 (***0.05***)
Urine						
Urine glucose (mM)	0.2 (±0.1)	0.4 (±0.2)	106.1 (±0.2)	2.79 (0.1)	677.3 (**9 × 10** ^**−12**^)	243 (**3 × 10** ^**−11**^)

a Mean ± SD, of *n* = 3 for each of the three groups.

b The significant changes (*p* < 0.05) are shown in bold.

^c^LDL, low-density lipoprotein; CREA, creatinine; MCH, mean corpuscular hemoglobin.

### Characterization of Metabolome and Lipidome

**FIGURE 2:**
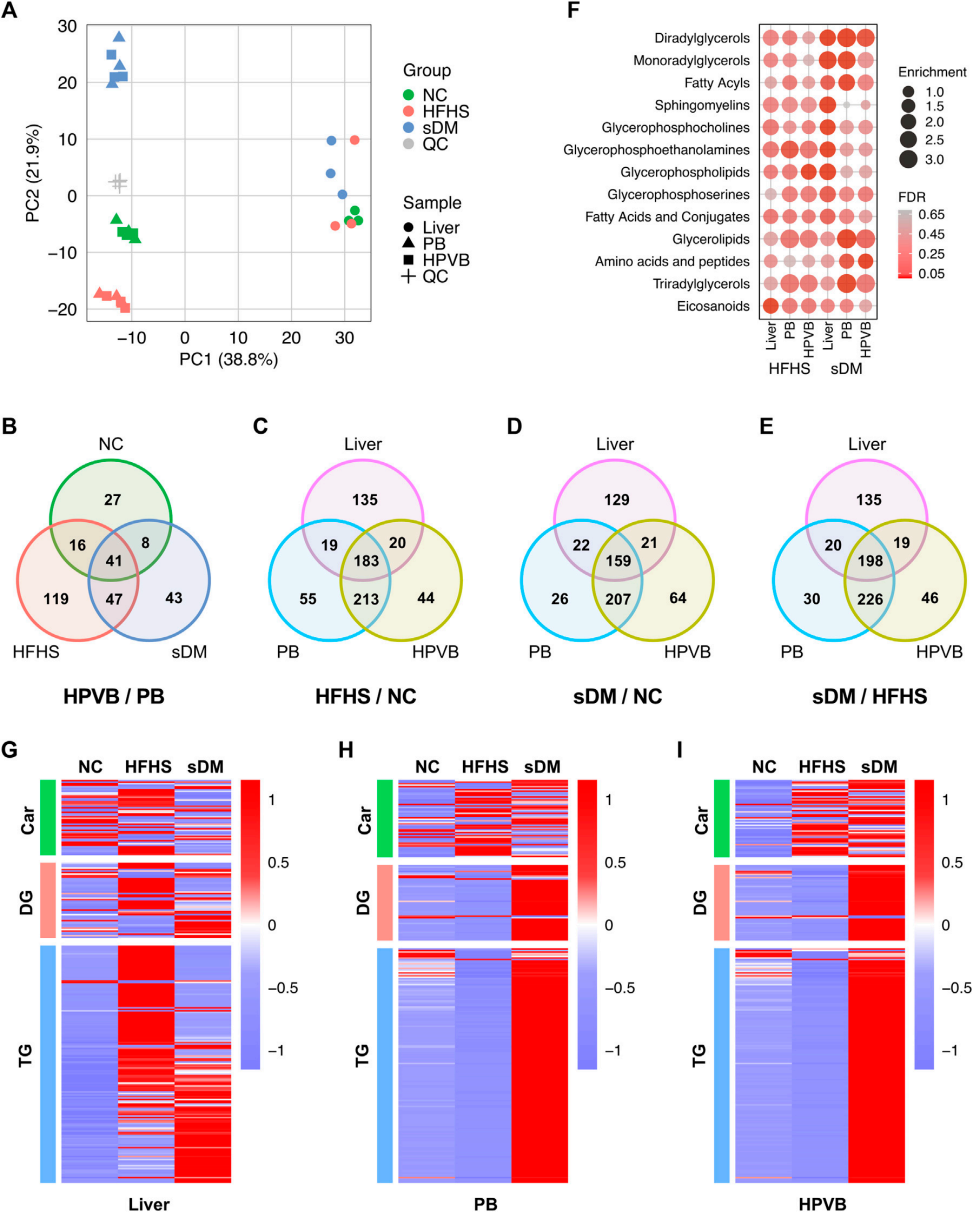
The comparison among the metabolomics data. **(A)** Principal component analysis of the 1,082 metabolites identified from all 27 samples and 4 QC samples. **(B)** The metabolites of significantly different levels between the PB and HPVB samples of each group. **(C–E)** The metabolites of significantly different levels between the HFHS and NC groups **(C)**, the sDM and NC groups **(D)**, and the sDM and HFHS groups **(E)**. **(F)** The enriched classes of molecules found from the metabolites altered in the HFHS and sDM groups. **(G–I)** The inter-group changes of acylcarnitines (Car), diacylglycerols (DG), and triacylglycerols (TG) in the liver **(G)**, PB **(H)**, and HPVB **(I)** samples.

Using the UPLC-MS/MS workflow, we identified 1,082 metabolites from all 27 samples (Supplementary Table S2). Slight higher numbers of metabolites were detected from the serum samples. Principal component analysis (PCA) of all samples illustrated a significant difference between the circulating metabolome and that from the liver tissues (Figure 2A). Also, the blood metabolomes of all groups had specific fingerprints but relatively small variants between PB and HPVB samples were observed (Figure 2A). Consistently, higher levels of correlations were observed between blood samples and between liver samples, but not between any blood and the liver sample, indicating that the profiles of detected metabolites captured the signature of the whole metabolome in different samples (Supplementary Figure S1). The inter-group difference in serum metabolome was much larger than that between the PB and HPVB of the same group of macaques (Supplementary Figure S2). In the NC monkeys, the PB and HPVB metabolomes had a difference of only 92 metabolites (Figure 2B; Supplementary Figure S3). More metabolites varied between PB and HPVB in the HFHS (223) and sDM (139) groups (Figure 2B; Supplementary Figure S2). On the other hand, over 400 metabolites in either PB or HPVB varied with the disease condition and/or the switch of diet (Figures 2C–E; Supplementary Figure S4; Supplementary Table S2), indicating the significant difference in the metabolism of these three groups. The PB and HPVB metabolomes shared a majority of altered metabolites. Although the metabolites changed in the liver had relative unique profiles, over half of them also overlapped those altered in sera (Figures 2C–E; Supplementary Figure S4; Supplementary Table S2). The altered metabolites are mainly enriched in the various classes of lipids as well as amino acids and peptides (Figure 2F). The spontaneously developed diabetes mellitus caused significant changes in the liver, characterized by the altered lipids of diacylglycerols, monoacylglycerols, fatty acyls, sphingomyelins, glycerophosphocholines, glycerophosphoethanolamines, glycerophospholipids, and glycerophosphoserines. Intriguingly, only polyunsaturated fatty acids increased in the liver as well as the blood of the sDM monkeys, whereas elevated levels of saturated, mono- and polyunsaturated fatty acids were observed in the HFHS groups compared with the NC group (Supplementary Figure S5). Consistent with the lipid droplets found from the HFHS livers (Figure 1I), lots of di- and triacylglycerol species increased in the liver of HFHS groups (Figure 2G). Instead of accumulation in the liver, di- and triacylglycerols elevated in the blood of the sDM monkeys (Figures 2H,I), further evidenced the diabetes of these macaques. Accordingly, enhanced levels of bile acids were measured from the HPVB of both the HFHS and sDM groups (Supplementary Figure S6). However, as compared with the HFHS group, the sDM monkeys failed to increase some bile acid species, including hyodeoxycholic acid, a bile acid of importance in regulating glucose homeostasis (Zheng et al., 2021b). In contrast, the sDM subjects had higher levels of taurocholic acid and taurodeoxycholic acid in both the liver and HPVB, which was also observed in the plasma of human patients (Mantovani et al., 2021). These observations suggest that distinct metabolic signatures of sDM and HFHS monkeys can be revealed by metabolomics and lipidomics, although some tendencies of bile acid changes in the prediabetic models were similar to those in the monkeys of diabetes.

### RNA-Seq Analysis of the Liver

**FIGURE 3:**
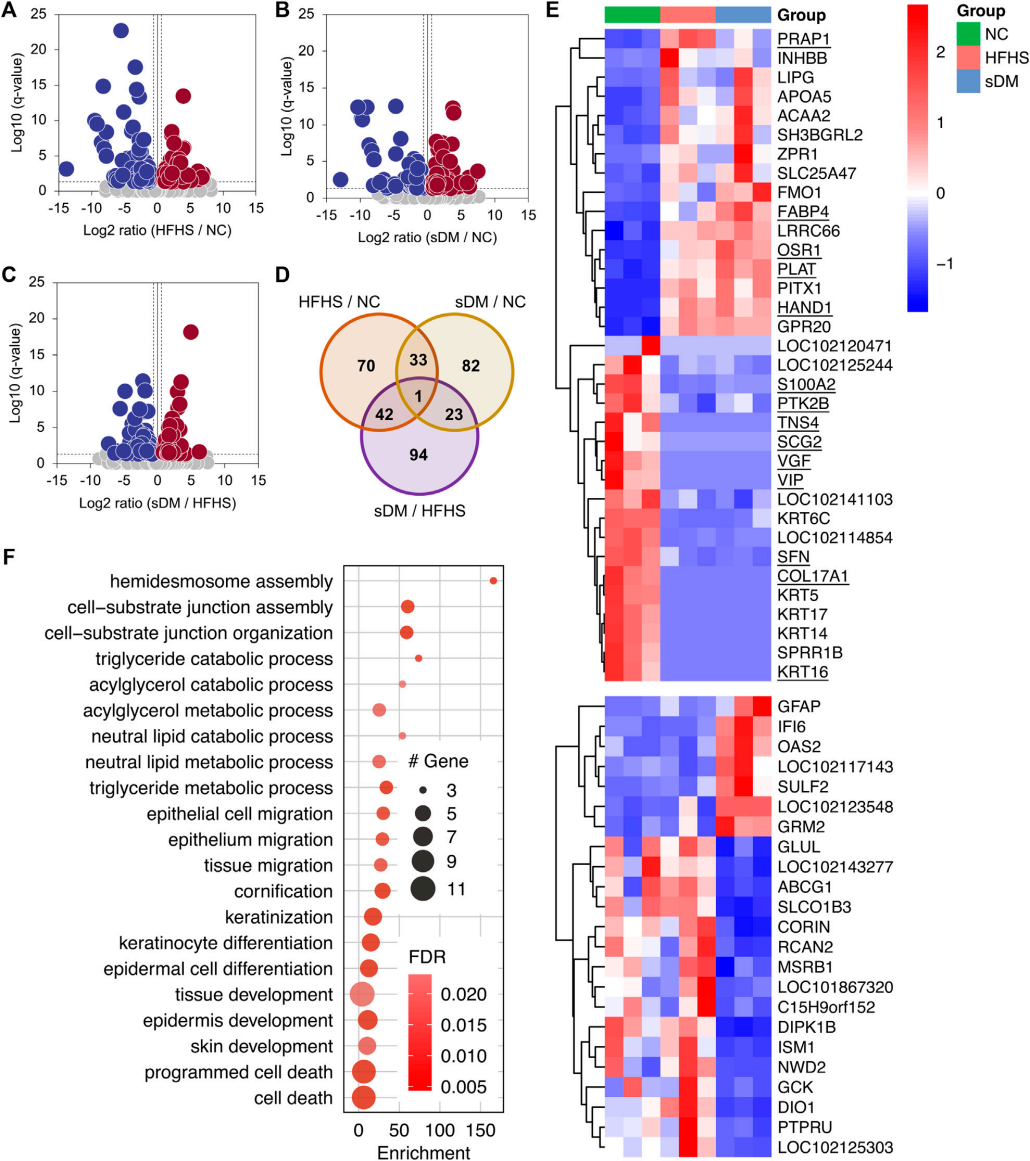
The expression profile in the livers of three groups. **(A–C)** The volcano plots of the genes differentially expressed between the HFHS and NC groups **(A)**, the sDM and NC groups **(B)**, and the sDM and HFHS groups **(C)**. **(D)** The altered transcripts between every two groups of the monkeys **(E)** The z-scored expression of the genes that significantly regulated commonly in both the sDM and HFHS macaques (upper panel) and only in the sDM group (lower panel). The genes that have known functions related to cell morphology and cell movement are underlined. **(F)** Fisher’s exact tested GO term enrichment (biological process) of the genes co-regulated in both sDM and HFHS groups.

The transcriptomics has profiled the expression of 18,841 genes, which were collapsed from over 46,000 transcripts, in the liver tissues of these monkeys (Supplementary Table S3). The detected transcriptome from each individual was similar in terms of both the total number of transcripts and their abundance distribution (Supplementary Figure S7). As compared with the NC group, the HFHS monkeys altered the expression of 146 (80 decreased and 66 increased) genes (Figure 3A). The numbers of down- and upregulated genes in the sDM group were 59 and 80, respectively (i.e., 139 in total; Figure 3B). Between the HFHS and sDM groups, 160 genes were different, out of which 89 and 71 showed higher levels in HFHS and sDM subjects, respectively (Figure 3C). Interestingly, the HFHS and sDM groups showed the largest difference in terms of the number of altered genes. (Figure 3D). The cross-comparison among the three groups demonstrated that these two groups shared only 33 altered genes (Figure 3D). The altered genes between HFHS versus NC groups and those between HFHS versus sDM groups showed a higher number in common, similar to the pattern observed from the comparison of the liver metabolome (Figure 2E). The genes altered in the HFHS groups consisted also of the ones participating in the metabolic and signaling processes of lipoprotein particles, cholesterol, and fatty acids; meanwhile, very-low-density lipoprotein particle remodeling was observed in sDM monkeys rather than HFHS group (Supplementary Figure S8). Focusing on the spontaneous DM, we further compared the genes that commonly changed in both the HFHS and sDM groups, and those regulated only in the sDM macaques (Figure 3E). The only genes differentially expressed in all three groups were PRAP1 (Figures 3D,E), which was recently reported as a lipid-binding protein promoting lipid absorption (Peng et al., 2021). As expected, many genes merely changed in the sDM group, such as SULF2, GFAP (Pang et al., 2017), ABCG1 (Daffu et al., 2015), GCK (Haeusler et al., 2015), ISM1 (Liu et al., 2019), and CORIN (Pang et al., 2015) (Figure 3E), have been associated with diabetes. The significant alterations of these genes observed only from the sDM groups but not the HFHS groups were consistent with the mild DM-like symptoms found from these monkeys (Figure 1). We performed a Gene Ontology (GO) enrichment analysis of the genes commonly regulated in both HFHS and sDM groups (Figure 3F). In accordance with the alterations in the metabolome, these regulated genes enriched in the cellular processes related to lipid metabolism. Additionally, the genes regulated in both HFHS and sDM groups included also the ones participating in the development of the extracellular matrix. The genes commonly regulated in both HFHS and sDM groups included a bunch of genes encoding proteins functioning in the lipid metabolism, such as LIPG, APOA5, ACAA2, and FABP4 (Figure 3E). Taken together, these results demonstrated the similarity in lipid metabolism between the sDM and HFHS groups, as those observed from the metabolomes (Figure 2). However, the expression of some known marker genes in the prediabetic macaques was still more similar to that in the healthy monkeys after 15 months of intake of extra fat and sugar.

Surprisingly, structural proteins and related regulators accounted for nearly a quarter (8 out of 33) of the genes altered in both the sDM and HFHS groups. These eight genes included keratins (KRT 5/6C/14/16/17), cell-matrix adhesion protein Collagen alpha-1 (XVII) chain (COL17A1), and cytoskeleton-binding regulatory proteins Tensin-4 (TNS4) and Cornifin-B (SPRR1B) (Figures 3E,F). In addition, 14 commonly regulated genes were known for their functions related to cell morphology and cell movement. They were upregulated genes HAND1, OSR1, FABP4, PLAT, and PRAP1, and the downregulated ones PTK2B, S100A2, SFN, TNS4, SCG2, VIP, KRT16, VGF, and COL17A1. These results suggested that the morphological changes in the liver caused by the HFHS diet were likely similar to that in the diabetes liver, which might be related to the pathogenesis of DM.

### Quantitative Proteomics of the Liver

**FIGURE 4:**
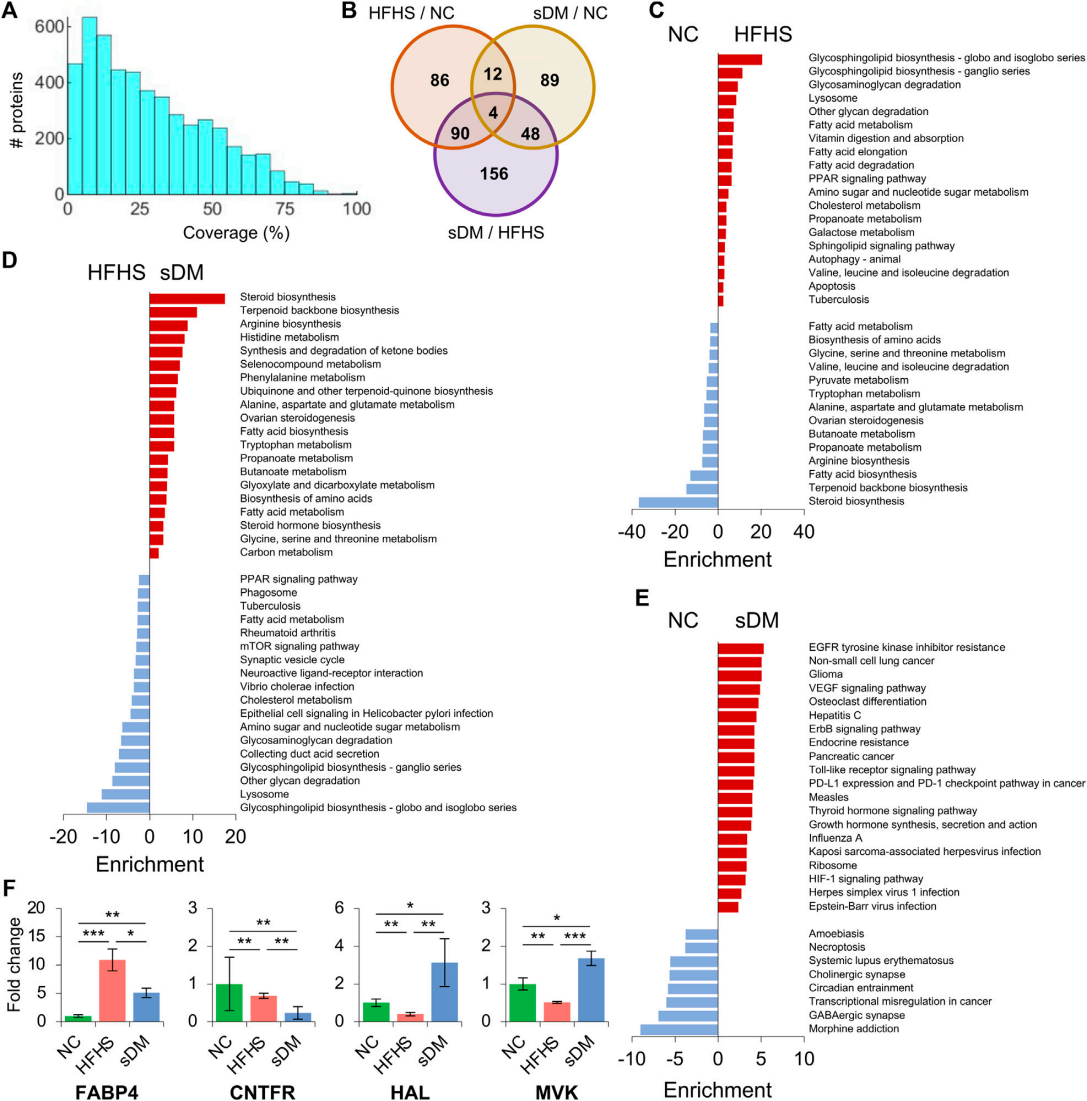
Proteomic profiling in the liver of the three groups. **(A)** The histogram of sequence coverage of all identified proteins. **(B)** The numbers of significantly changed proteins between the groups. **(C–E)** KEGG pathway enrichment of the altered proteins between the NC and HFHS groups **(C)**, between the sDM and HFHS groups **(D)**, and between the sDM and NC groups **(E)**. **(F)** The fold changes of the four differentially expressed proteins among all three groups. FABP4; Fatty acid binding protein 4; CNTFR, Ciliary neurotrophic factor receptor; HAL, Histidine ammonia-lyase; MVK, Mevalonate kinase. Mean ± SD are shown. *, *p* < 0.05; **, *p* < 0.01; ***, *p* < 0.001.

Proteomics identified approximately 5000 proteins from the liver tissues (Supplementary Table S3). The average sequence coverage was 28.6% and about 99% of them (4,900 out of 4,954) were identified with at least one unique peptide (Figure 4A; Supplementary Table S4). Similar to that observed in the transcriptome, these proteomics data also showed that the highest alteration between the HFHS and sDM macaques, and the largest number of regulated proteins in common between the cross-comparisons of HFHS versus NC as well as HFHS versus sDM (Figure 4B). Individually, the HFHS diet triggered the alterations of 192 proteins, out of which 71 and 121 were down- and upregulated, respectively (Supplementary Figure S9A). Also, the spontaneous DM resulted in the changes in 153 proteins, 51 decreased and 102 increased, in the liver (Supplementary Figure S9B). Between the HFHS and sDM groups, we observed 298 proteins of various abundance, consisting of 169 down- and 129 upregulated ones (Supplementary Figure S9C). When comparing the differential proteins from these two pair-wise contrasts, it was found that the HFHS-enhanced metabolic pathways were largely overlapped when referred to the NC (Figure 4C) and sDM groups (Figure 4D). The commonly upregulated glycan degradation pathways, PPAR signaling pathway, fatty acid metabolism, sugar metabolism, and cholesterol metabolism indicated the significant changes in the hepatic proteome caused by the food composition. However, the proteins in the sDM livers that were differentially expressed in comparison to the proteins in the NC and the HFHS groups enriched in the distinct pathways (Figure 4E). In contrast to the NC macaques, the altered proteins in the sDM livers functioning in various singling pathways, whereas the comparison between the HFHS and sDM groups mainly highlighted the enrichment of changed proteins in biosynthetic pathways (Figure 4D). Four proteins were differentially expressed in all three groups (Figures 4B,F). Fatty acid binding protein 4 (FABP4) increased dramatically in both HFHS and sDM groups and showed the highest level in the former (Figure 4F). FABP4 has been reported as a marker protein for type 2 diabetes and a protein target for inflammatory diseases. CNTFR, the receptor for ciliary neurotrophic factor (CNTF), was reduced in both the HFHS and sDM groups. Preview studies have also proposed CNTF as a candidate agent for the therapy of diabetes complications (Ma et al., 2018). Both histidine ammonia-lyase (HAL) and mevalonate kinase (MVK) decreased in the HFHS group but elevated in the sDM groups (Figure 4F). Mevalonic acid is the precursor for cholesterol synthesis, in which MVK is a key enzyme. Epidemiological studies have demonstrated that inhibiting the production of mevalonic acid increases the risk of developing type 2 diabetes.

### Cross-Omics Insights Into the Alterations of the Liver

**FIGURE 5:**
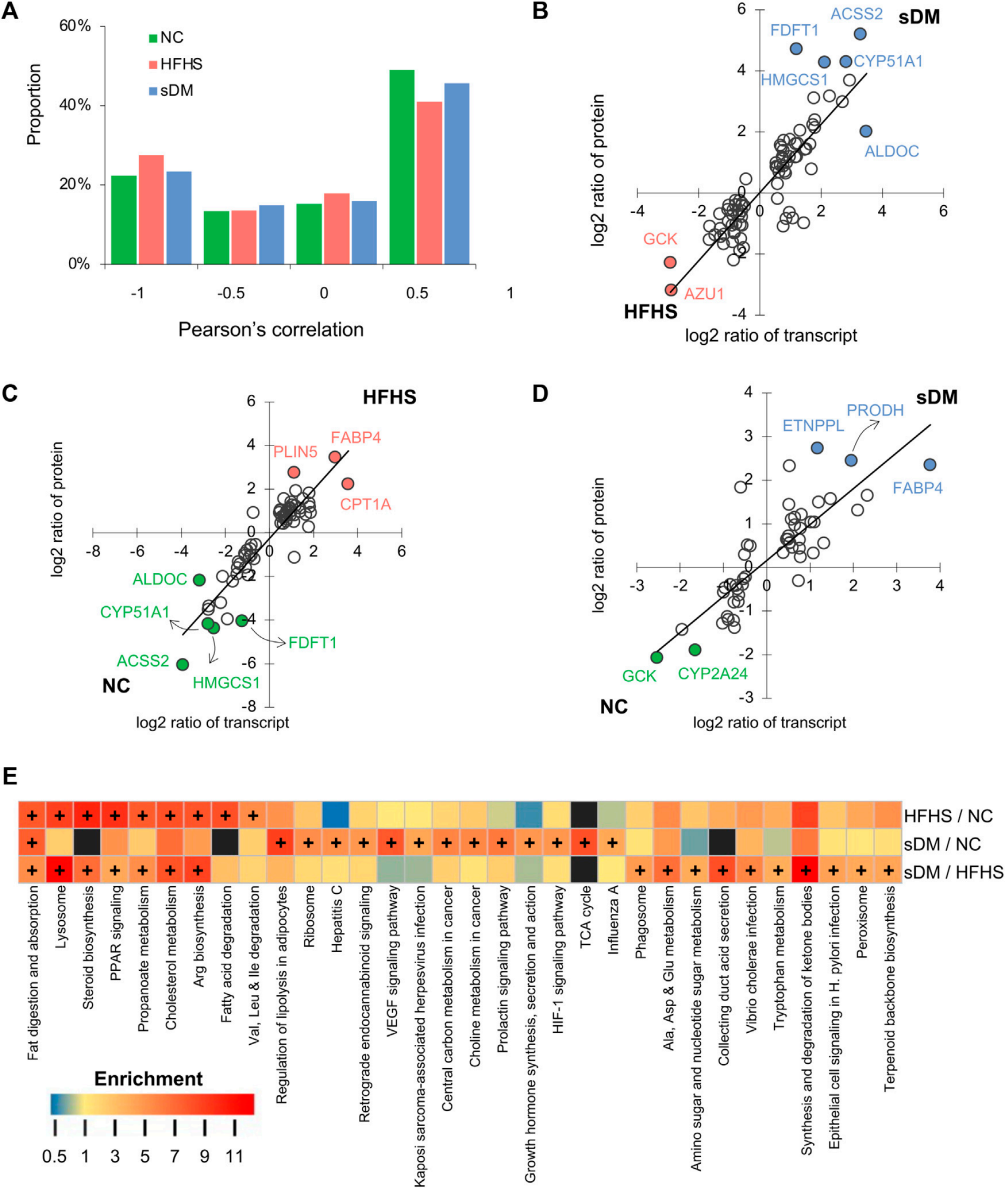
Joint analysis of the multiomics data. **(A)** The proportion of genes whose proteins and transcripts showed positive (> 0.5), negative (< −0.5), or no correlation (between −0.5 and 0.5). **(B–D)** the comparisons of log2 ratios of protein and transcript levels of differential genes for sDM versus HFHS **(B)**, HFHS versus NC **(C)**, and sDM versus NC **(D)**. The black lines show the bisects y = x. **(E)** The pathway enrichment analysis using the metabolomics and proteomics data jointly. Plus symbol (+) annotates the enriched pathways of significance.

To find the common features of HFHS and sDM, we performed cross-omics analyses and provided insights into pathological changes in macaque livers. For the genes whose transcripts and their encoded proteins were both detected from transcriptomic and proteomic approaches, respectively, we calculated the Pearson correlation between the transcript and protein of each gene across all samples of each group and found positive correlations (Pearson’s correlation coefficient >0.5) for about a half of them (Figure 5A). For the significantly regulated genes, a strong correlation between the changes in transcripts and proteins was found when every two groups of macaques were compared (Figures 5B–D). The consistency showed the changed protein abundance caused by the pathogen of DM and/or the switch to HFHS food were predominately regulated *via* gene expression, which was captured in our data precisely. Both transcriptomic and proteomic data supported upregulation of FABP4 in both HFHS and sDM groups and showed the highest level in the former (Figures 4F, 5C,D). FABP4 is one of the biomarkers that has been associated with both type 1 and 2 DM (Rodríguez-Calvo et al., 2019; Xiao et al., 2021). The abundance of phosphotransferase (GCK) in the sDM livers was much lower than those in either the NC or HFHS groups (Figures 5B,D), evidencing the repression of hepatic glycolysis in DM. The HFHS diet triggered the downregulation of fructose-bisphosphate aldolase C (ALDOC), a key enzyme in both glycolysis and gluconeogenesis pathways (Figures 5B,C). Two enzymes that were involved in the mevalonate pathway, 3-hydroxy-3-methylglutaryl coenzyme A synthase (HMGCS1) and farnesyl-diphosphate farnesyltransferase 1 (FDFT1), were also increased in the HFHS livers. Together with the consistent changes in MVK (Figure 4F), these data indicated the suppression of cholesterol synthesis in the HFHS monkeys. Acyl-CoA synthetase short chain family member 2 (ACSS2) that promotes fat storage was also lower in the HFHS groups, suggesting the downregulation of this protein in response to the increased intake of fat. We then conducted the joint analysis of the metabolomics and proteomics data. As expected, all three groups had differentially regulated fat digestion and absorption pathway (Figure 5E). Also, switching to the HFHS diet resulted in significant changes in some mo lipid metabolism-related pathways, such as steroid biosynthesis, PPAR signaling, propanoate metabolism and cholesterol metabolism. As a disease group, the sDM group had significant changes in the regulatory and disease-related pathways, including the HIF-1 signaling pathway, prolactin signaling pathway, and growth hormone synthesis, secretion and action pathway. On the other hand, the divergence between the HFHS and sDM groups included both metabolic and regulatory pathways. Taken together, our data demonstrated that the HFHS diet-fed macaques showed many molecular alterations in common with the changes in the DM ones. However, the accumulated changes that can cause cellular responses in oxygen homeostasis and inflammation might be of importance in the development of diabetes.

## Discussion

The present study included the relative abundance of both transcripts and proteins in the liver and relative levels of metabolites and lipids in the liver, PB, and HPVB sera from the same individuals, providing data suited to conducting correlation analysis at a systems level. Multi-omic data provides comprehensive profiling of biological samples. Proteins are functional macromolecules that arguably participate in almost all cellular processes. The amounts of this machinery are modulated through transcriptional regulation as well as other types of regulatory mechanisms such as post-transcriptional regulation and protein degradation. From our data, we found good correlations between transcripts and proteins for over 50% of genes (Figure 5A). Although only a few subjects were included in our study, this consistency strongly supported alterations of these genes, including FABP4, GCK, ALDOC, HMGCS1, FDFT1, and ACSS2. For a big proportion of these genes, their functions related to DM have been documented to some extent, which further validated our results. Previous reports have evidenced that the correlations between mRNA transcripts and the corresponding proteins are not always held for a certain number of genes (Maier et al., 2009; Vogel and Marcotte, 2012). Although positive correlations were found for nearly half of the genes, no or even negative correlated cases existed in this dataset. These cases provide the candidates for the studies of DM-caused protein regulation behind gene transcription. Moreover, the changes in various classes of lipids (Figure 2) and the alterations in the transcripts (Figure 3) and proteins functioning in lipid signaling and metabolism (Figures 4, 5) also indicated the consistency of our data.

The switch of diet, to our surprise, caused larger numbers of altered molecules in the metabolome, lipidome, transcriptome, and proteome. The majority of changes in the HFHS monkeys were not as same as those in the sDM group (Figures 3D, 4B). This unique profile was primarily characterized by the alterations in the pathways related to lipid metabolism, indicating the hepatic response to the extra intake of fat and sugar. As compared with both NC and HFHS groups, the sDM macaques showed responses in the genes functioning in disease-related pathways and regulatory pathways (Figures 4C,D, 5E). Metabolic inflammation is an essential feature of type 2 diabetes. This feature was indicated by the changes in the pathways related to inflammation signaling and oxygen hemostasis, such as including the HIF-1 signaling pathway, prolactin signaling pathway, growth hormone synthesis pathway, as well as the signaling pathways for EGRF and VEGF, in our data (Figures 4E, 5E). Studies in animal models have shown that diet-induced oxidative stress and inflammation play a crucial role in the pathogenesis of type 2 diabetes (Shoelson et al., 2006). Diet-induced animal models of DM have long been employed in the studies of diabetes (Kleinert et al., 2018; Engel et al., 2019). Our data demonstrated that 1.25 years of HFHS diet feeding in *Macaca fascicularis* did not cause strong signals of inflammation and oxidative stress, resulting only in mild obesity and prediabetic symptoms (Figure 1). The disagreement observed from our data has also suggested that it is of importance to measure these factors related to the inflammatory and stressed statues in the studies of diet-induced models for DM.

Nonetheless, the alterations common in the sDM and HFHS groups had also been detected. This similarity was mainly the alterations in the metabolism of lipids and fatty acyls (Figures 3F, 5E), characterized by the increased lipid levels (Figure 2) and the upregulation of FABP4 (Figures 4, 5). FABP4 (also known as adipocyte Protein 2 or aP2) is a lipid-binding carrier that regulates fatty acid trafficking, which is also involved in linking lipid metabolism with innate immunity and inflammation (Furuhashi and Hotamisligil, 2008). FABP4 is a secreted protein that is widely expressed in adipocytes, macrophages, and endothelial cells (Xu and Vanhoutte, 2012). Under normal conditions, the expression of this gene is low in the liver, which was confirmed by the low reads of the NC group (Supplementary Table S3). Our transcriptomic and proteomic data consistently supported the increase of this protein in both the sDM and HFHS groups (Figures 5C,D). These results, especially the elevated levels of the FABP4 transcript, indicated the upregulation of this gene in the liver of both diabetic and prediabetic macaques. FABP4 regulates hepatic production of glucose (Cao et al., 2013), and the mice with FABP4 deficiency have a lower risk for obesity-induced insulin resistance and type 2 DM (Tuncman et al., 2006). Elevated levels of circulating FABP4 have been proposed as a marker of DM-related syndrome (Rodríguez-Calvo et al., 2019), with a pro-diabetic impact in the process of diabetes in the pancreas (Xiao et al., 2021). The upregulation of FABP4 expression in the liver might also have a role in the development of DM. Furthermore, an animal study has demonstrated that Sirtuin 1 (SIRT1) regulates FABP4 secretion from the white adipose (Josephrajan et al., 2019). Sirtuins are signaling proteins functioning in metabolic regulation. The levels of SIRT1 are involved with the insulin sensitivity of cells and, therefore, associated with non-alcoholic fatty liver disease and DM (Martins, 2018b). Although SIRT1 showed no significant changes in its expression, SIRT5, another member of the Sirtuin protein family, increased 1.5 fold in the sDM group (Supplementary Tables S3, S4). SIRT5 has been suggested to be involved in energy metabolism (Verdin et al., 2010; Park et al., 2013). We may speculate that FABP4 regulates hepatic lipid metabolism of the sDM macaques in a sirtuin-dependent manner. It is worthy of further investigation that the roles of hepatic FABP4 and sirtuins in the development of DM. As a secreted protein, the increase of FABP4 protein in the liver might also partially attribute to the alterations in other tissues such as adipose tissue. The interaction between FABP4 of various sources is likely an intriguing and important topic that should be addressed.

Intriguingly, the gene set whose expression was similarly changed in our prediabetic and diabetic monkeys contained a lot of genes functioning in cell migration and cellular/extracellular structure (Figure 3). The structural proteins have critical regulatory functions in sensing and signaling. We have found significant changes in keratin expression. Keratins are a family of fibrous proteins forming intermediate filaments, which have been found to participate in the insulin-mediated regulation of glucose metabolism (Roux et al., 2016). The downregulated keratins (KRT 5/6C/14/16/17) are all skeleton proteins of epithelial cells. It has been demonstrated that insulin resistance disrupts epithelial repair and tissue remodeling in the liver through impairing local cellular crosstalk (Manzano-Núñez et al., 2019). Expression of COL17A1, a transmembrane collagen protein important in maintaining the linkage between the intra- and extracellular structural elements (Franzke et al., 2005), was not detectable in the sDM macaques (Supplementary Table S3). A previous study has also reported that reduction of COL17A1 promotes the migration of keratinocytes (Tasanen et al., 2004). These results, together with the enriched alterations in cell migration (Figure 3F) the EGFR signaling (Figure 4E), indicated the impeded epithelial reparation in the liver of sDM macaques, resulting in defects in wound healing and liver injury (Brem and Tomic-Canic, 2007). The regulation of these genes in the HFHS group showed a similar tendency, suggesting that these responses caused by the HFHS diet might occur earlier than the systematic oxidative stress and inflammation. Thus these alterations possibly mediated the HFHS diet-induced diabetes, which is worth further investigation in the future.

This multi-omics dataset from the NHP of spontaneous diabetes mellitus is capable of serving as a resource for various types of diabetes researches. NHPs are used in research into human diseases due to their genetic similarity to *Homo sapiens*. Macaques, including those in *Macaca fascicularis,* have contributed to the studies of DM for decades (Yasuda et al., 1988; Hansen, 2010; Bauer et al., 2011; Harwood et al., 2012; Pound et al., 2014; Havel et al., 2017). Recently, other groups have also reported multi-omics datasets from monkeys of spontaneous type 2 DM (Lei et al., 2020), and monkeys fed with a high-fructose diet (Cox et al., 2021). Our work has added an additional database for scientists in the field. This present study covers the quantification of both serum metabolome and molecular profiling of the liver from both the spontaneous type 2 DM monkeys and HFHS-fed monkeys grown and measured in parallel, providing a database of quality in studies on the pathological impacts of HFHS in DM. Human studies, such as that on clinical trials, would likely have fewer respects of data in general. The changes in the gene expression of the macaque liver related to the alterations in the blood can sever as a reference for this type of investigation.

## Conclusion

In the present work, we conducted a multi-omics analysis to compare the molecular alterations in the liver, HPVB, and PB of macaques of spontaneous Type 2 DM, and ones of prediabetic symptoms caused by high-fat and high-sugar diet. Our data indicate that protein FABP4, a lipid-binding protein that has a crucial role in DM, was upregulated in the liver of spontaneously occurred diabetes and HFHS-induced diabetes, evidenced by elevation in both hepatic mRNA and protein levels. Furthermore, we have also revealed that at the prediabetes stage, the HFHS diet could cause malfunctions in the epithelial cell morphology and migration, similar to the alterations in the DM monkeys. These defects may impair liver reparation and promote DM. Also, our work provided a new multi-omic dataset from NHP that is likely to benefit other DM studies.

## Data Availability

The raw data supporting the conclusion of this article will be made available by the authors, without undue reservation. The raw proteomic data and the identification and quantification results of MaxQuant are available at ProteomeXchange Consortium (https://www.ebi.ac.uk/pride/) with the identifier (PXD025863). RNA-seq data and quantification results have been deposited at ArrayExpress (https://www.ebi.ac.uk/arrayexpress/) with the identifier (E-MTAB-10493). The metabolomics data have been deposited to Metabolomics Workbench (https://www.metabolomicsworkbench.org/), which is accessible with the identifier (ST001935).
