# Supplementary material for: Multi-Omics Comparison of the Spontaneous Diabetes Mellitus and Diet-Induced Prediabetic Macaque Models
Source: Front Pharmacol. 2021 Nov 22;12:784231. doi: 10.3389/fphar.2021.784231 (PMC8645867; doi:10.3389/fphar.2021.784231)
Supplement: Supplementary file 3 [file Presentation1.PPTX]

## Slide 1
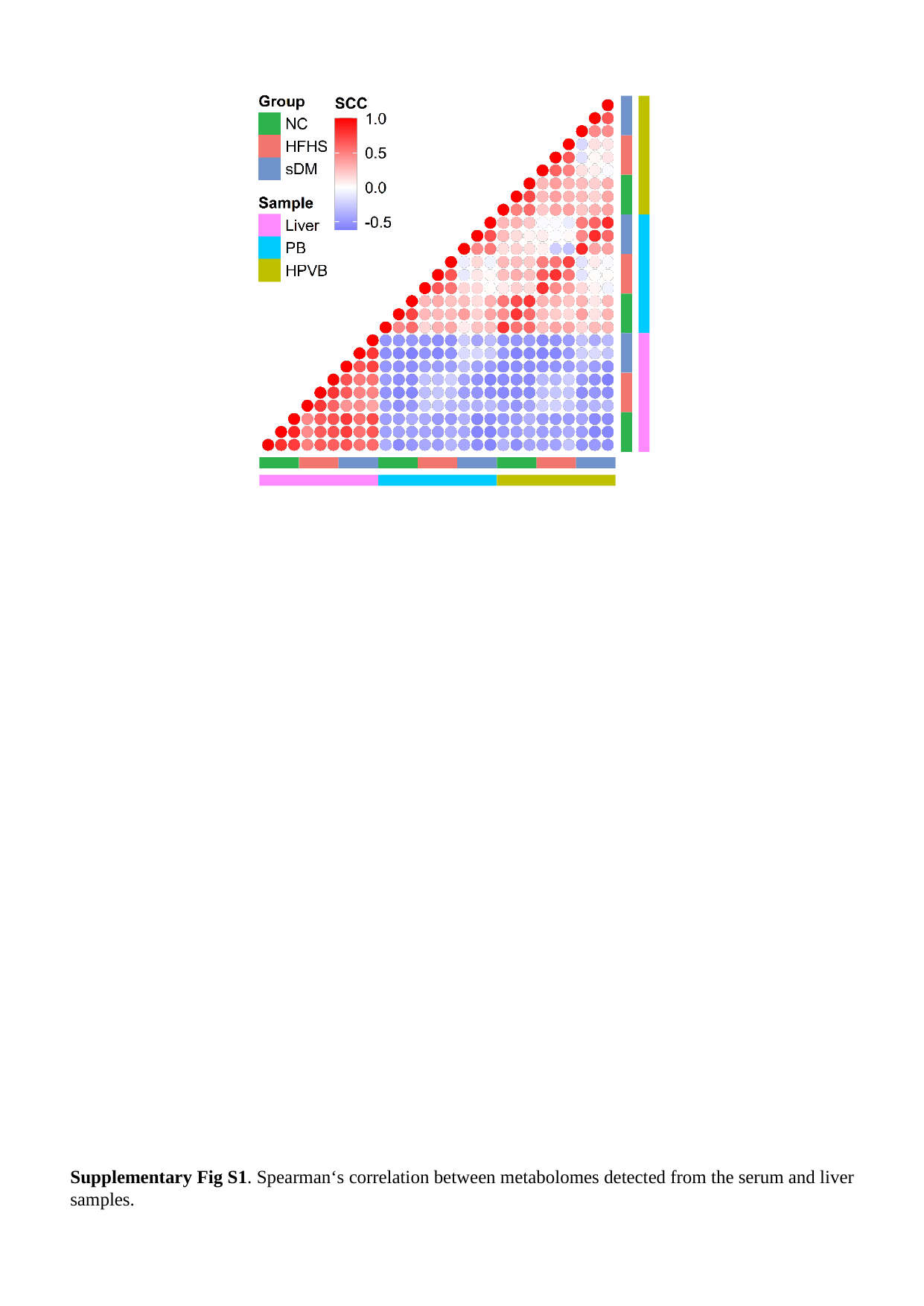

Supplementary Fig S1. Spearman‘s correlation between metabolomes detected from the serum and liver samples.

## Slide 2
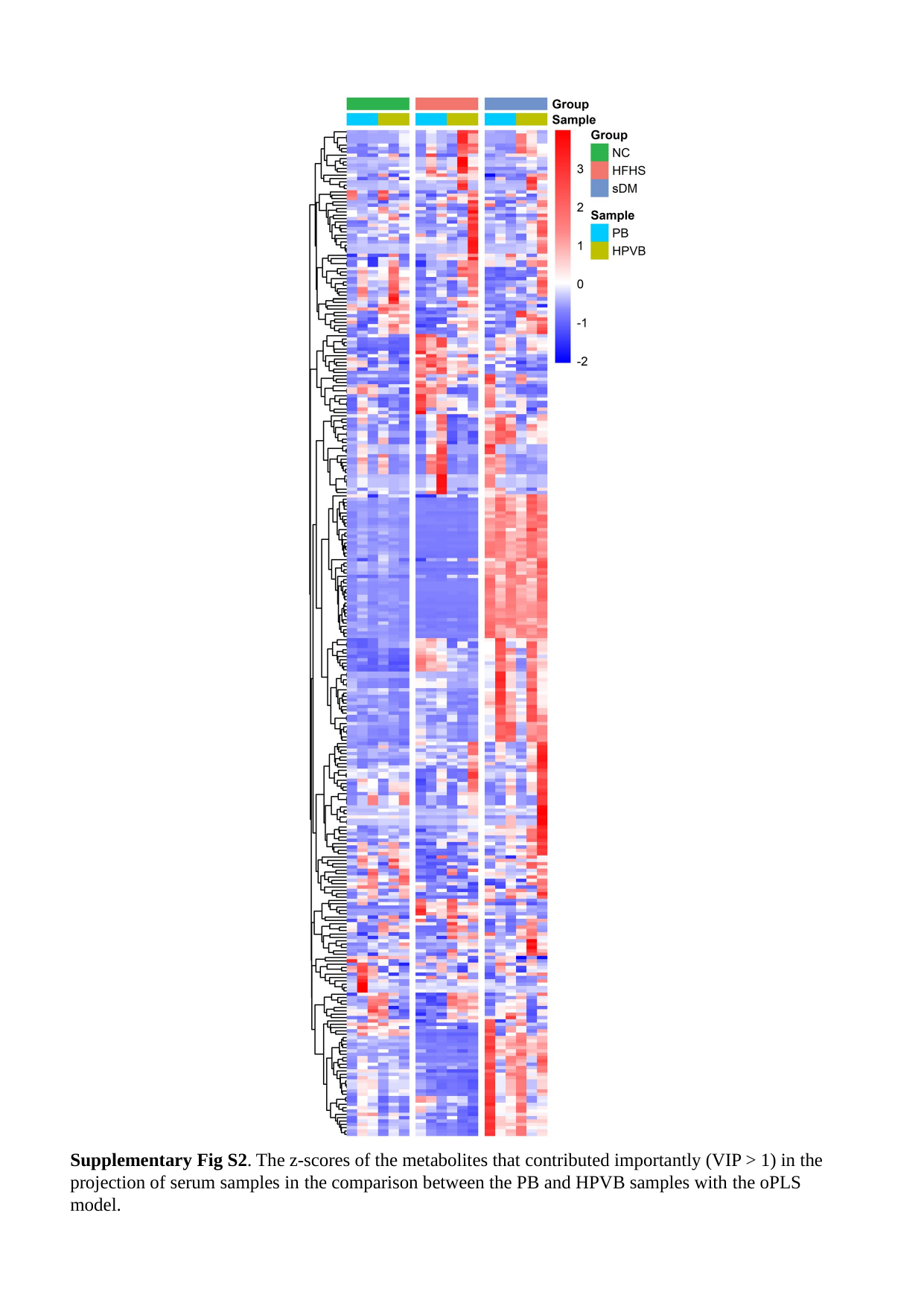

Supplementary Fig S2. The z-scores of the metabolites that contributed importantly (VIP > 1) in the projection of serum samples in the comparison between the PB and HPVB samples with the oPLS model.

## Slide 3
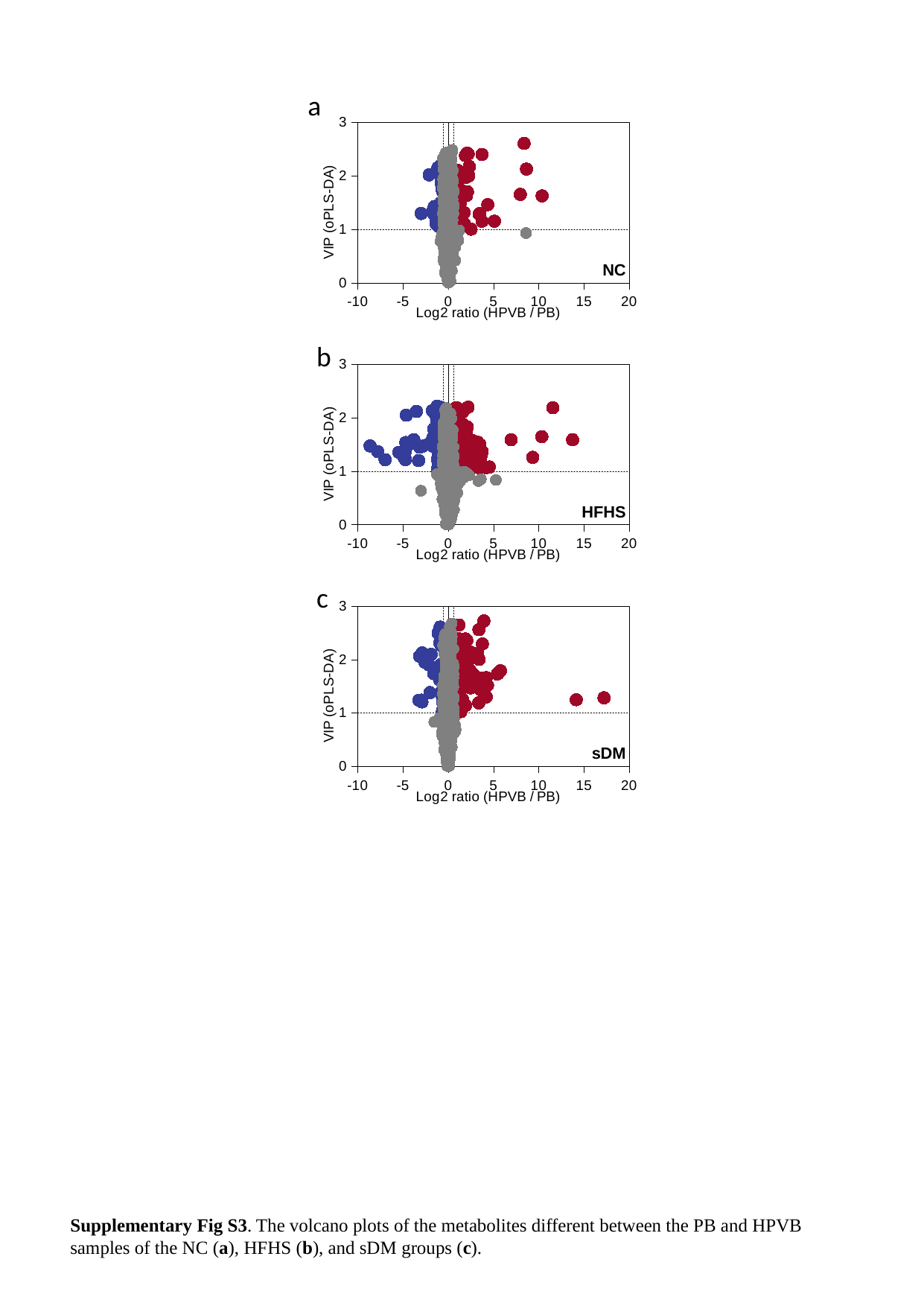

a
### Chart
| Category | Normal.vip | | | | | | |
|---|---|---|---|---|---|---|---|NC
b
### Chart
| Category | HFD.vip | | | | | | |
|---|---|---|---|---|---|---|---|HFHS
c
### Chart
| Category | Diabetes.vip | | | | | | |
|---|---|---|---|---|---|---|---|sDM
Supplementary Fig S3. The volcano plots of the metabolites different between the PB and HPVB samples of the NC (a), HFHS (b), and sDM groups (c).

## Slide 4
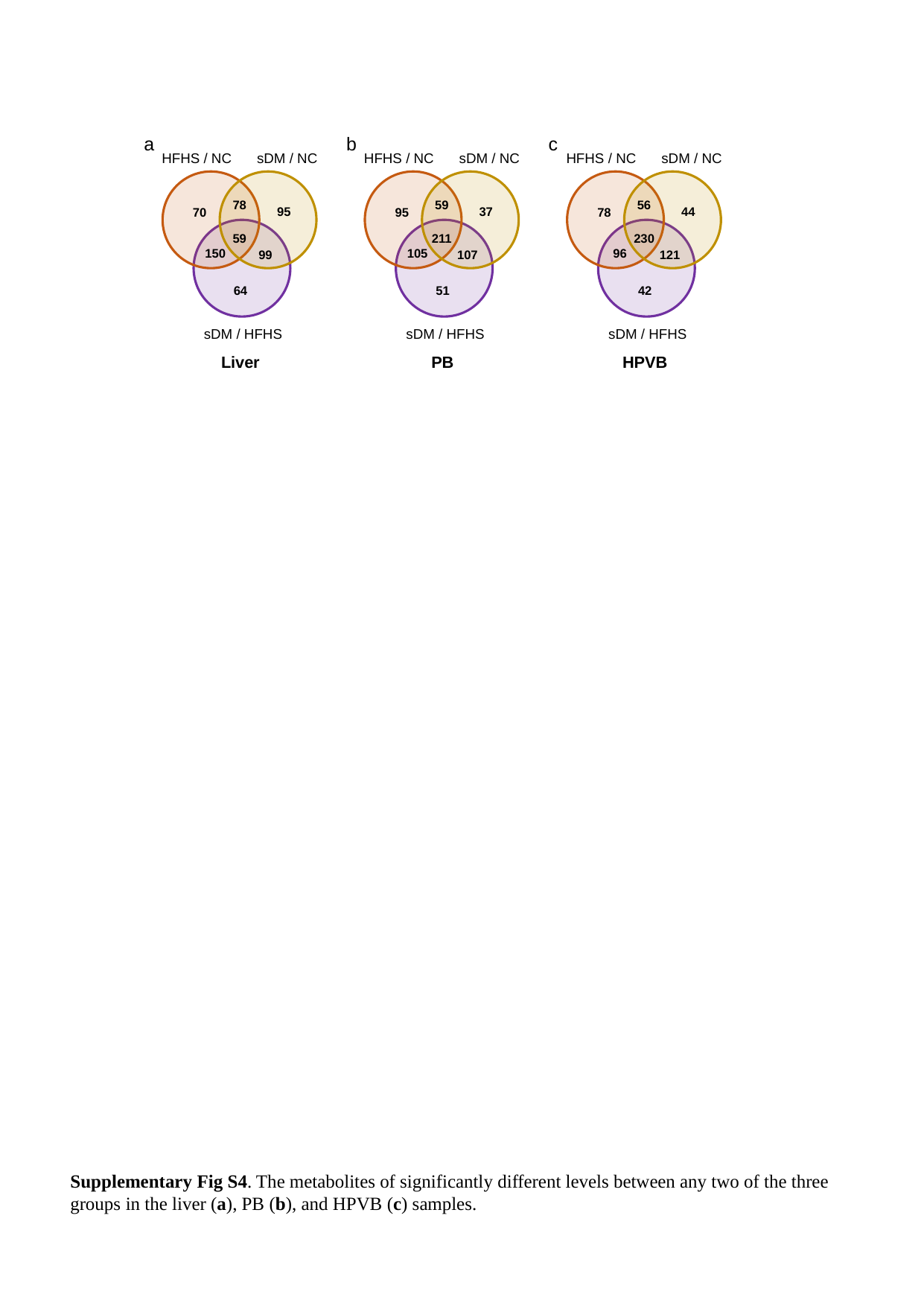

a
HFHS / NC
sDM / NC
78
95
70
59
150
99
64
sDM / HFHS
Liver
b
HFHS / NC
sDM / NC
59
37
95
211
105
107
51
sDM / HFHS
PB
c
HFHS / NC
sDM / NC
56
44
78
230
96
121
42
sDM / HFHS
HPVB
Supplementary Fig S4. The metabolites of significantly different levels between any two of the three groups in the liver (a), PB (b), and HPVB (c) samples.

## Slide 5
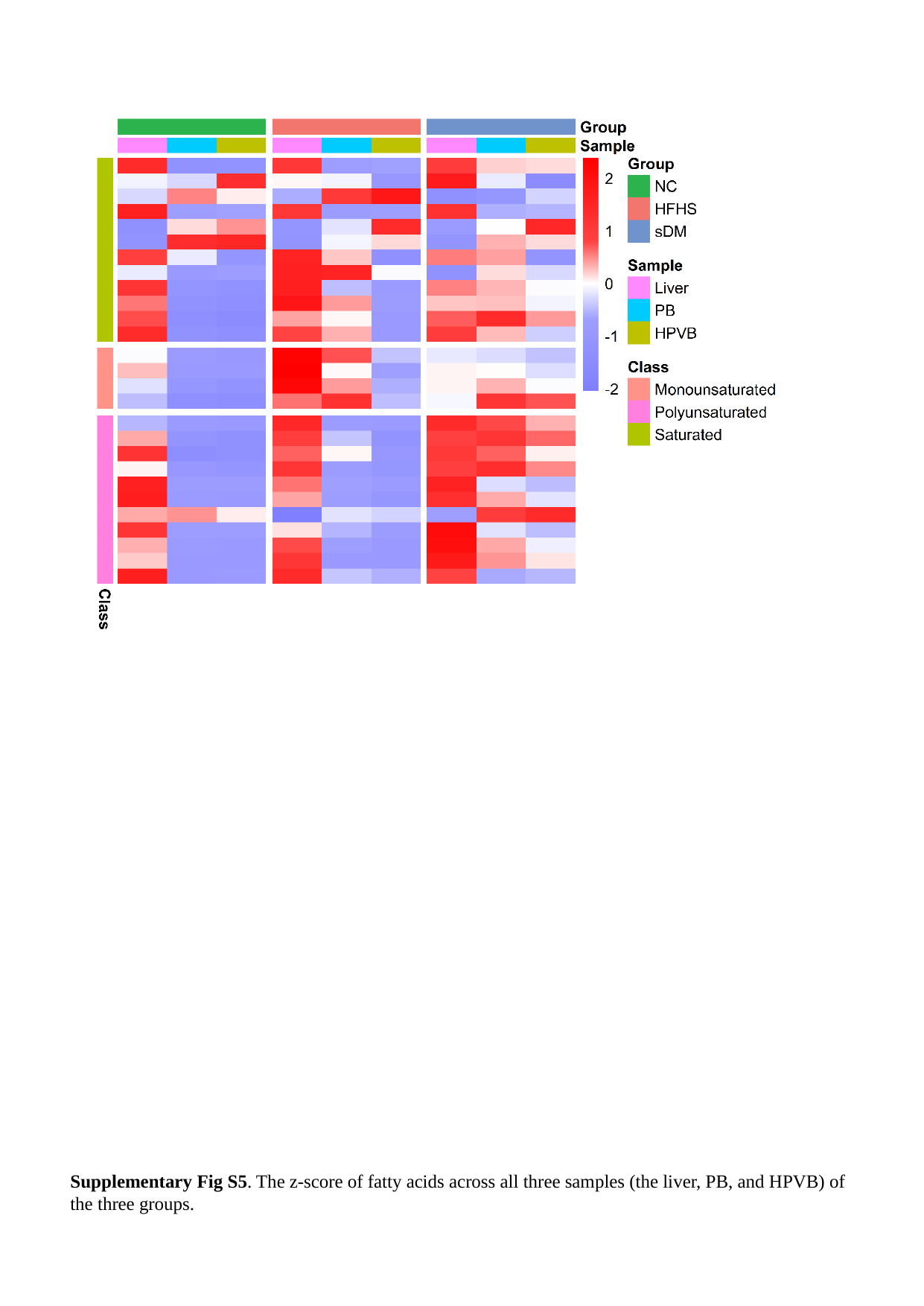

Supplementary Fig S5. The z-score of fatty acids across all three samples (the liver, PB, and HPVB) of the three groups.

## Slide 6
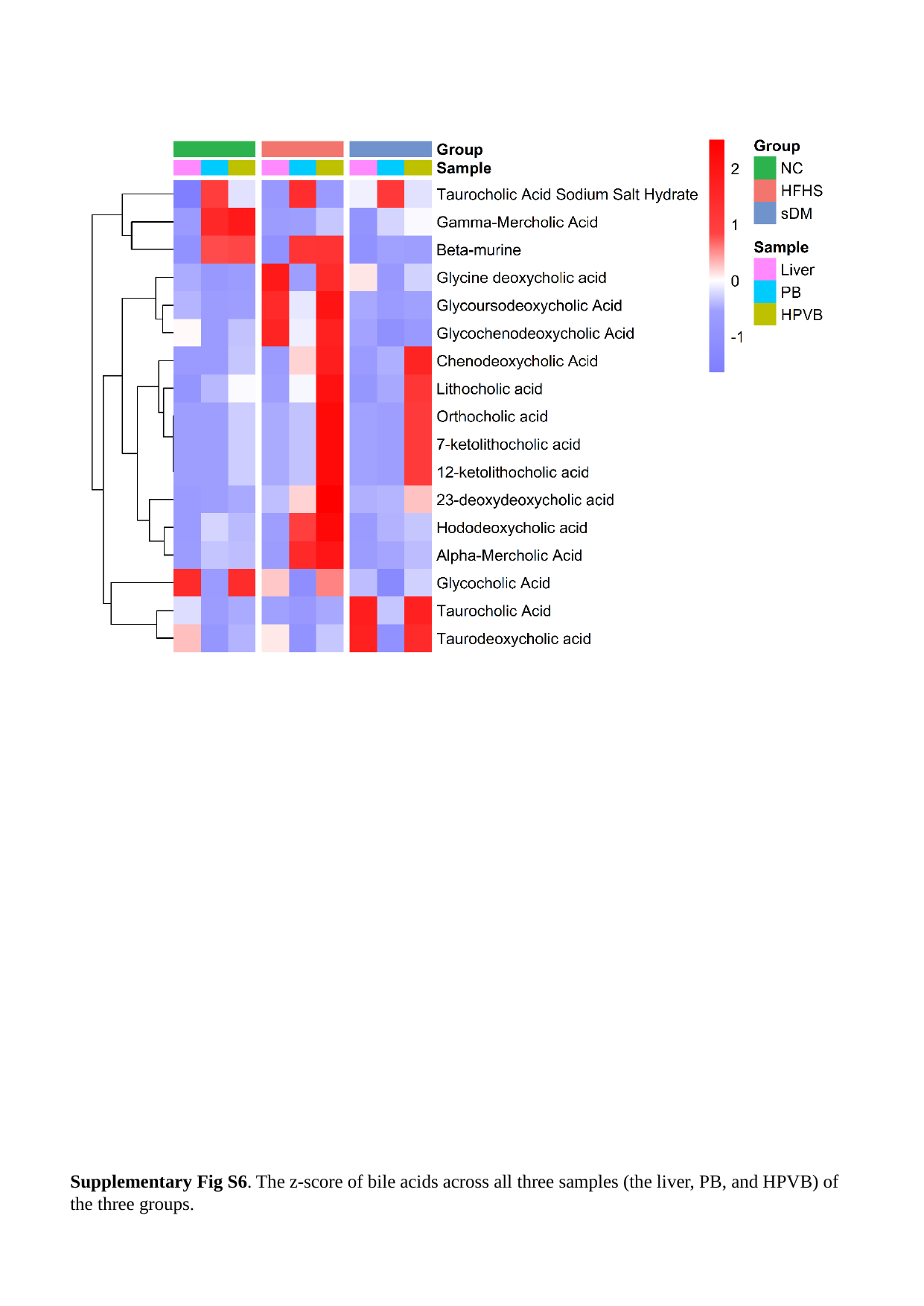

Supplementary Fig S6. The z-score of bile acids across all three samples (the liver, PB, and HPVB) of the three groups.

## Slide 7
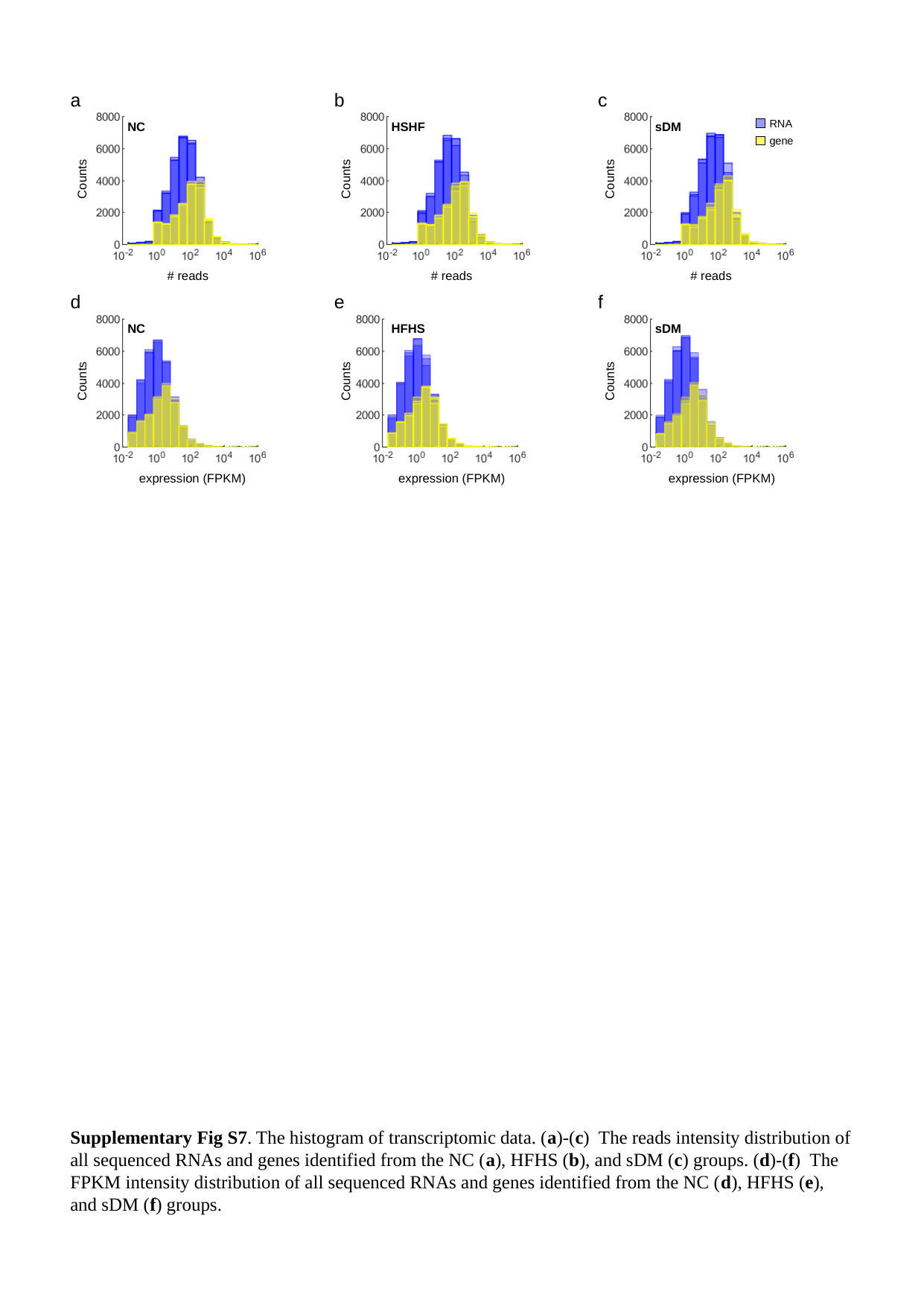

a
NC
Counts
# reads
b
c
sDM
Counts
# reads
RNA
gene
HSHF
Counts
# reads
d
NC
Counts
expression (FPKM)
e
HFHS
Counts
expression (FPKM)
f
sDM
Counts
expression (FPKM)
Supplementary Fig S7. The histogram of transcriptomic data. (a)-(c)  The reads intensity distribution of all sequenced RNAs and genes identified from the NC (a), HFHS (b), and sDM (c) groups. (d)-(f)  The FPKM intensity distribution of all sequenced RNAs and genes identified from the NC (d), HFHS (e), and sDM (f) groups.

## Slide 8
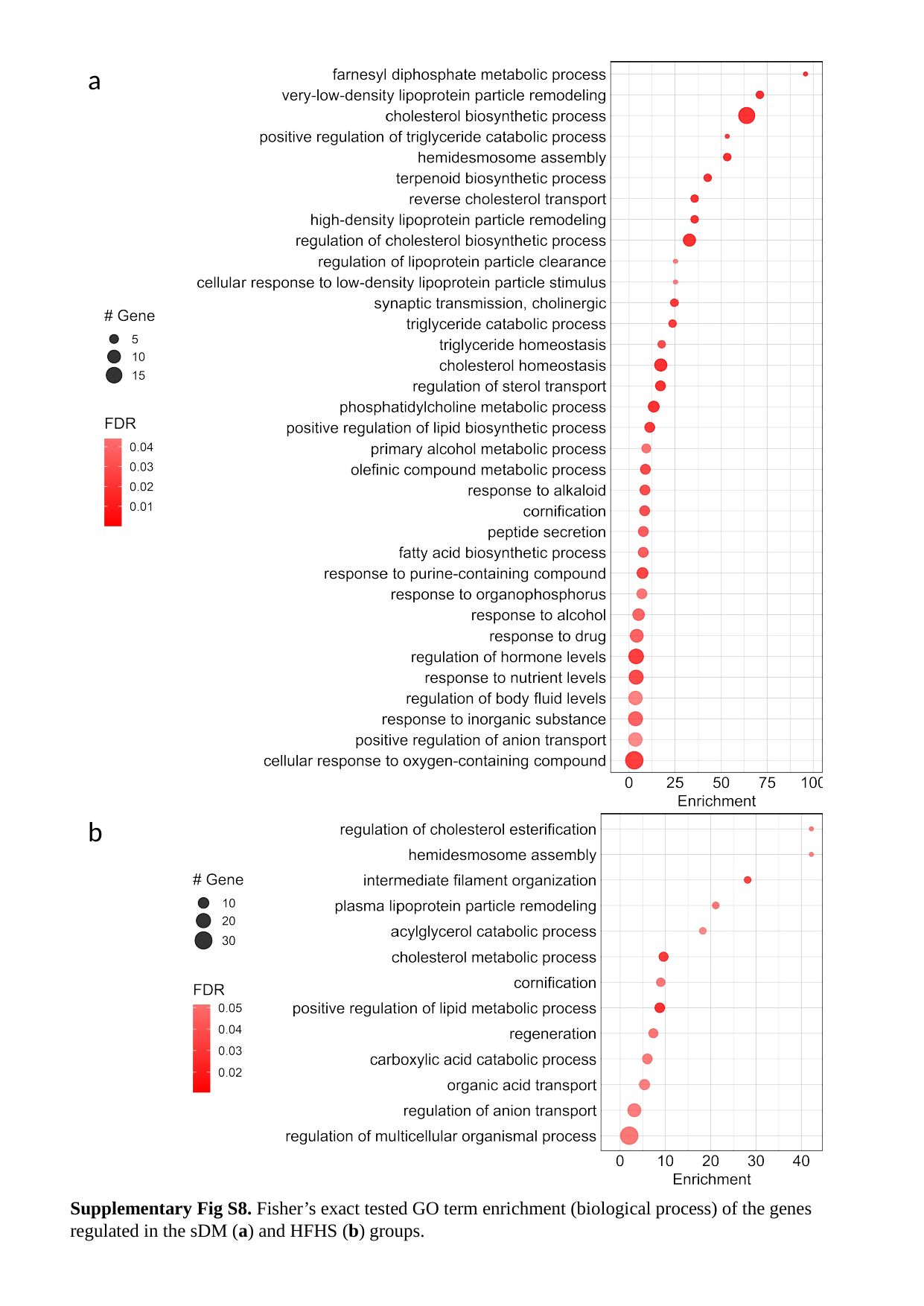

a
b
Supplementary Fig S8. Fisher’s exact tested GO term enrichment (biological process) of the genes regulated in the sDM (a) and HFHS (b) groups.

## Slide 9
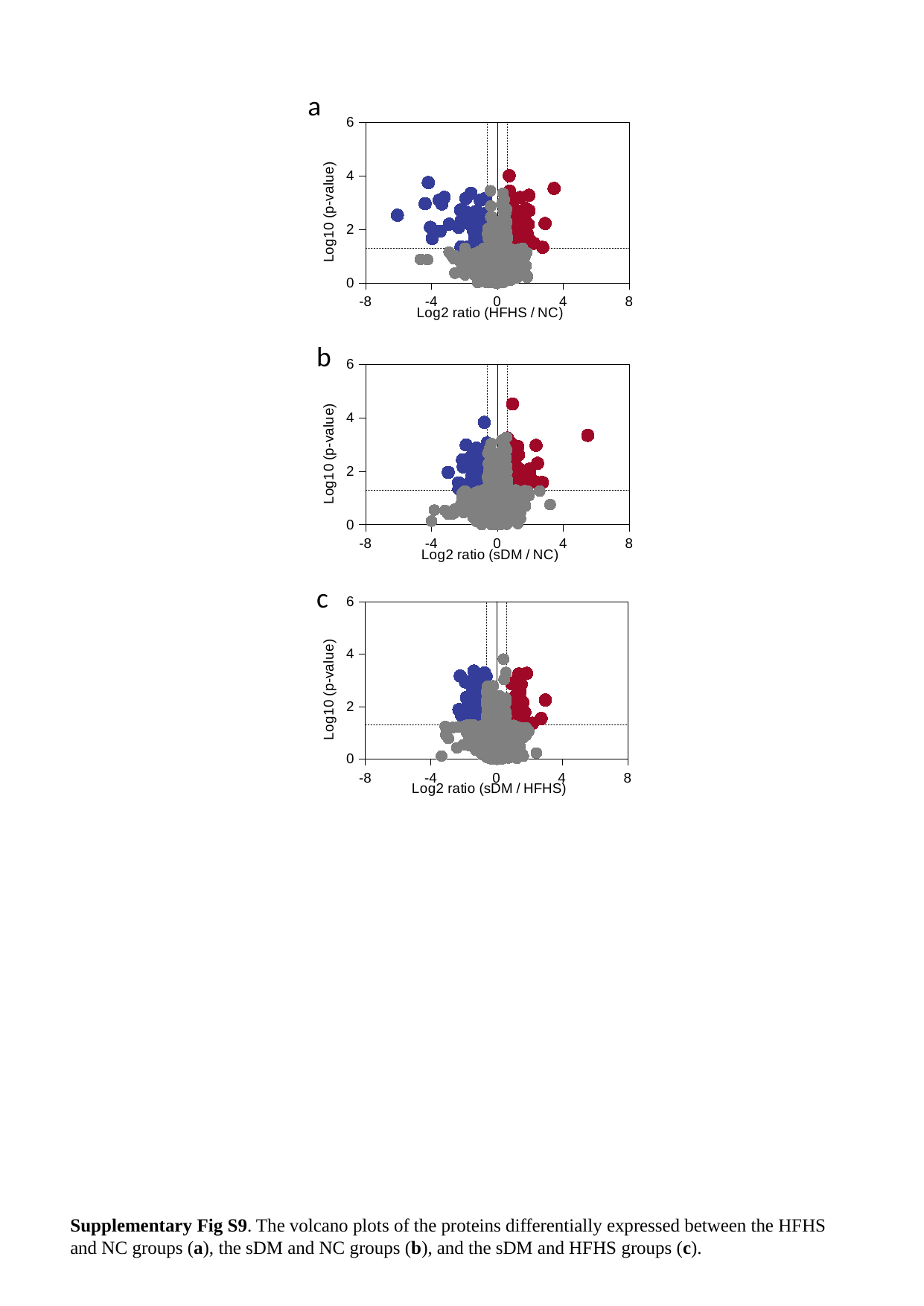

a
### Chart
| Category | -log10q | | | | | | |
|---|---|---|---|---|---|---|---|b
### Chart
| Category | -log10q | | | | | | |
|---|---|---|---|---|---|---|---|c
### Chart
| Category | -log10q | | | | | | |
|---|---|---|---|---|---|---|---|Supplementary Fig S9. The volcano plots of the proteins differentially expressed between the HFHS and NC groups (a), the sDM and NC groups (b), and the sDM and HFHS groups (c).
